# The Political Economy of the World Health Organization Model Lists of Essential Medicines

**DOI:** 10.1111/1468-0009.70001

**Published:** 2025-02-27

**Authors:** KRISTINA JENEI

**Affiliations:** ^1^ London School of Economics and Political Science

**Keywords:** essential medicines, universal health coverage, World Health Organization, access to health care, politics, pharmaceutical economics

## Abstract

**Policy Points**

- The World Health Organization (WHO) Model Lists of Essential Medicines (EML) aims to select clinically beneficial and cost‐effective medicines that ought to be prioritized by health systems based on the priority needs of their populations. However, the rapid evolution within the pharmaceutical sector toward complex, high‐priced medicines has challenged WHO decision making in recent years, as evidenced by earlier literature demonstrating inconsistencies in the application of decision criteria and recommendations.
- Proposed solutions to these challenges focus on technical aspects of the program, such as refining the quality of evidence in applications, improving the connection with guidelines, and using evidence assessment frameworks. Yet, earlier literature has not examined the political challenges that the WHO—as a global health organization—has encountered during the past 20 years. This article examines these challenges by reviewing documents and interviewing stakeholders involved with the WHO EML decision making.
- A diverse range of stakeholders shape the process to select medicines, each with different interests (e.g., protecting commercial interests versus advocating for access) and ideas (the role of the WHO EML in indirectly resulting in lower prices versus safeguarding low‐ and middle‐income countries from catastrophic expenditure). A lack of data and financial and human resources inhibits evaluation of the impact of the EML and exacerbates the influence of external actors, including which products are reviewed and how they are recommended. As a result, a degree of inconsistency has emerged, both in recommendations and in the concept of essential medicines.

**Context:**

The World Health Organization (WHO) Model Lists of Essential Medicines (EML) aims to help countries select medicines based on the priority needs of their populations. However, rapid evolution within the pharmaceutical sector toward complex, high‐priced medicines has challenged WHO decision making, leading to inconsistent decisions. The purpose of this paper is to investigate how political factors impact the WHO EML.

**Methods:**

Document review and semistructured interviews of diverse stakeholder groups with direct experience with the WHO EML, either as stakeholders involved with WHO EML processes (e.g., selection of medicines, observers) or external applications (*n* = 29). Donabedian's structure–process–outcome framework was combined with the Three I's framework (ideas, interests, and institutions) to understand how political factors shape the WHO EML.

**Findings:**

The concept of essential medicines evolved from an original focus on generic medicines in resource‐constrained countries to include complex, high‐priced therapeutics also relevant to high‐income nations. The WHO has never explicitly addressed whom its decisions are for. Some believe the Model Lists have a “symbolic” price‐lowering mechanism, whereas others do not (e.g., the pharmaceutical industry concerns to profitability). This tension has led to different ideas and interests driving the EML. A lack of data and human resources inhibits evaluation and exacerbates the influence of external actors. A degree of inconsistency has emerged in the concept and recommendations of essential medicines.

**Conclusions:**

The current debate about the role of the WHO EML centers on the question whether the Model Lists ought to include complex, high‐priced medicines. However, this research demonstrates that challenges may have roots deeper than amending decision criteria. At the core of this issue is the role of the list. Defining a strategic vision for the WHO EML, refining decision criteria, and increasing institutional support would align interests, good processes, and, ultimately, contribute to positive societal health outcomes.

At the center of the essential medicines concept is the World Health Organization (WHO) Model Lists of Essential Medicines (EML; or Model Lists), a program that aims to select clinically beneficial and cost‐effective pharmaceuticals that ought to be prioritized by health systems worldwide. Since its first publication in 1977, the WHO EML have reinforced the concept of essential medicines through the biannual publication of the Model Lists. The Model Lists were designed to guide national policies and aid countries to develop national essential medicines lists (NEMLs).^1^ The WHO EML intend to promote rational use of drugs, improve access to affordable treatments, and inform procurement and supply chain management.^1^

Over the past 50 years of the history of the Model Lists, the pharmaceutical ecosystem has evolved and with it the concept of essential medicines. The first 25 years of the WHO EML was characterized by an increasing scope of the Model Lists, as well as the political and economic tensions of determining which medicines are considered “essential.” Originally, affordability was a “major criterion” for inclusion, resulting in a list of mostly generic medicines—a compromise to members of the pharmaceutical industry given concerns about the impacts of the rationalization in high‐income countries.^2^ Against the backdrop of the increasing burden of the HIV/AIDS epidemic, the exclusion of highly effective but high‐priced antiretroviral drugs incited widespread indignation from nongovernmental organizations (NGOs),^167^, ^188^ which prompted changes to the selection process. The official decision criteria were amended in 2001 to state that costs and patent status cannot be reasons for exclusion from the WHO EML. Over the following two decades, several high‐priced and effective medicines were added,^4^ widening the relevance of the essential medicines concept to include those that are highly relevant to high‐income countries.^5^, ^6^, ^7^ The result is a concept that has shifted slightly over time to encompass the difficulties health systems are facing worldwide,^8^, ^9^ sparking a debate on the scope and purpose of the Model Lists.^10^

The rapid evolution within the pharmaceutical sector toward complex, high‐priced medicines has contributed to several inconsistencies in WHO decision making in recent years. The inclusion of several high‐priced, complex medicines such as trastuzumab for breast cancer, sofosbuvir for hepatitis C, brand name sodium‐glucose cotransporter 2 inhibitors, and insulin analogs reflects their positive impact on patient outcomes. However, the exclusion of other similarly complex and expensive treatments for cancer despite exceeding WHO‐specified thresholds for clinical benefit and safety has raised questions about the consistency of the WHO's decision criteria across therapeutic areas, particularly when balancing efficacy, public health relevance, and cost.^10^, ^11^, ^12^ Medicines listed on the WHO EML for rare diseases have tripled over the past two decades, contradicting an original stance against their inclusion on the WHO EML given a mandate to focus on “the priority needs of the population.”^13^, ^14^ Economic evidence has been assessed and weighed inconsistently across recommendations.^3^ Recently, the World Federation of Hemophilia stated that the WHO EML was “moving backwards, not forwards” given the inclusion of medicines for bleeding disorders that contradicted international hemophilia guidelines.^15^, ^16^ Collectively, these concerns raise questions about procedural transparency in the selection of essential medicines and undermine the WHO's credibility as a normative organization promoting universal access to essential medicines.^11^, ^17^, ^18^

The purpose of this paper is to investigate how political factors impact the WHO EML. Proposed solutions for improving consistency of the WHO EML processes have focused solely on ameliorating technical aspects of the selection procedures. For example, suggestions include assessing applications through evidential frameworks, such as the Grading of Recommendations, Assessment, Development, and Evaluations (GRADE),^19^ strengthening the link between the WHO EML and guideline development,^20^ raising the evidential standards of applications,^19^, establishing a separate committee for the assessment of clinical benefit and economic criteria,^11^ or refocusing the Model Lists on a subset of countries.^10^ Acknowledging the importance of technical considerations in medicine selection, these solutions overlook the political realities of pharmaceuticals, the WHO, and the global health ecosystem. The concept of essential medicines is not a  indisputable monolith but a norm constructed by several stakeholders, their interests, and ideas over time. The ascension of essential medicines to global acceptability is not the result of natural progression but rather the outcome of a competition among the pharmaceutical industry, the WHO, academics, and NGOs to redefine the concept after their own priorities.^21^, ^22^, ^23^, ^24^, ^25^ Yet, very little attention has been given to these aspects in the literature, particularly over the past two decades, during which pharmaceutical innovation has undergone substantial transformation. Therefore, the aim of this study is to explore the factors outside evidence appraisal that have contributed to inconsistencies in recommendations for essential medicines.

## Methods

### Theoretical Framework

**Figure 1:**
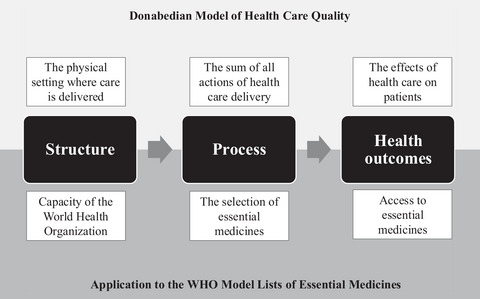
The Donabedian Model of Structure, Process, and Outcomes Model Applied to the WHO EML WHO, World Health Organization.

This research combines the Donabedian model with the Three I's (ideas, interests, and institutions) framework to understand how political factors impact the quality of health care programs, such as the WHO EML. In 1966, Avedis Donabedian introduced a widely used conceptual model for examining the quality of health care delivery.^26^ According to the model, information about quality can be derived from three domains: structure, process, and outcomes (Figure 1). “Structure” describes the physical and organizational factors relating to the setting in which care is delivered. This includes the physical environment, equipment, data, and human resources. Structure is critical because it “increases the likelihood of good processes, and a good process increases the likelihood of good outcomes.”^26^ The process is the sum of all actions for which health care is delivered. In the context of the WHO EML, this refers to the technical procedures for selecting essential medicines. The outcome relates the effects of health care on patients. In the Donabedian model, this could include changes to health status, patient satisfaction, health‐related quality of life, or death. When applied to the WHO EML, outcomes could be reflected in increased access to essential medicines or, more broadly, in the WHO's mandate to “promote health, keep the world safe, and serve the vulnerable.”^27^ Outcomes are often interpreted as the most important indicators of quality given the goal of health care programs and health outcomes.

Donabedian developed the model to be flexible and applied in a variety of ways. Most directly, the conceptual model has been applied to assess the quality of a single health care intervention within a hospital setting, such as neonatal resuscitation^28^ or COVID‐19 responses,^29^ and to improve disease management for treating certain chronic conditions, such as lupus.^30^ The model has been used outside direct quality improvement projects, such as mapping national strategies for digital health policies^31^ and evaluating undergraduate nursing education.^32^ More broadly, the model was the basis for large‐scale evaluation of health delivery, such as disability services across the state of New York.^33^

The objective of the paper is to understand how political factors impact the WHO EML. Therefore, a natural question arises: What is meant by political factors? “Politics” has been defined as “who gets what, when, and how.”^34^ Three of the most common factors included in political analyses are actors’ interests, their ideas and institutional factors that either exacerbate or suppress these influences. A framework, commonly referred to as the “Three I's” framework, brings these factors together to explain public policy processes.^35^ In this framework, interests are defined as the “agendas of societal groups, elected officials, civil servants, researchers, and policy entrepreneurs.”^36^ Interests are directly correlated with actors’ power to shape policy. Scholars have described the authority of private and nonactors in the development of global essential medicines policy, such as the pharmaceutical industry, academics, and NGOs.^23^, ^24^ Ideas are defined as “the content and strength of actors’ values and knowledge in the policy process.”^36^, ^37^ Put simply, ideas are the ways problems and solutions are framed by actors. The discourse and framing that shapes agendas has been described by politicians as “the most powerful thing on Earth.”^38^ Institutions are the “rules of the game”^39^—the formal and informal rules that shape how decisions are made and implemented. This might include legal frameworks, organizational rules, and less explicit cultural norms. The “Three I's” framework has been used extensively in health policy analyses, such as explaining health care reform in low‐ and middle‐income countries (LMICs),^36^ policy changes to the social sector in Ireland,^40^ and the formulation of international policy for HIV.^41^. Applied to the WHO EML, these categories include the WHO organizational setting, the processes for medicine selection, and the program outcomes (Figure 1).

Combining the Donabedian model of health care quality and the Three I's framework provides the main categories of health care programs to evaluate (structure, process, and outcomes) and the political factors to assess within each domain (ideas, interests, and institutions). In this sense, the paper examines how ideas and interests intersect with institutional factors in defining the WHO EML's structure, the ideas and interests of different stakeholders that intersect in defining the process of selecting medicines, and ideas and interests that influence the definitions of outcomes. This approach addresses a major limitation in health policy analyses, in which studies merely describe what happened and do not examine the dynamic interplay between several factors and their impact on outcomes.^42^

### Study Design and Data Collection

The study combines data from a document review and key‐informant interviews in an iterative study design. First, documents were collected through a targeted search of the WHO and other global health organizations and peer‐reviewed literature. Second, these documents were analyzed to understand gaps that were subsequently addressed with the key‐informant interviews. Interviews, document review, and analysis continued iteratively until saturation (the point at which further data do not produce new insights). A figure demonstrating the study design is included in Appendix 1.

### Document Review

Peer‐reviewed and policy materials regarding the WHO EML were collected, including the following: 1) WHO reports, meeting minutes, and guidance documents; 2) gray literature, including reports and documents published by other major global institutions involved in the access to medicines movement; and 3) secondary materials such as peer‐reviewed literature and media articles. Initially, materials outside the study period were included to capture the history of the essential medicines program. As the study continued, a targeted search for documents within the study period (2000‐2023) was conducted. WHO documents were used to create a timeline and identify critical actors for the interviews. These documents were identified through the WHO publications database^43^ by searching “essential medicines” and by reviewing references cited in relevant documents. Documentation from other organizations contextualized the WHO EML within global health development over the past two decades. These documents were found by targeted searches through organizational websites. A list of organizations queried is included in Appendix 2. Secondary materials, such as media articles and peer‐reviewed literature, provided commentary and additional discourse on essential medicines, including key challenges and opportunities. These articles were found through a systematic literature search of six databases. The details of the search terms are included in Appendix 3. A sample of literature was consulted but not referenced directly.^44^, ^45^, ^46^, ^47^, ^48^, ^49^, ^50^, ^51^, ^52^, ^53^, ^54^, ^55^, ^56^, ^57^, ^58^, ^59^, ^60^, ^61^, ^62^, ^63^, ^64^, ^65^, ^66^, ^67^, ^68^, ^69^, ^70^, ^71^, ^72^, ^73^, ^74^, ^75^, ^76^, ^77^, ^78^, ^79^, ^80^, ^81^, ^82^, ^83^, ^84^, ^85^, ^86^, ^87^, ^88^, ^89^, ^90^, ^91^, ^92^, ^93^, ^94^, ^95^, ^96^, ^97^, ^98^, ^99^, ^100^, ^101^, ^102^, ^103^, ^104^, ^105^, ^106^, ^107^, ^108^, ^109^, ^110^, ^111^, ^112^, ^113^, ^114^, ^115^, ^116^, ^117^, ^118^, ^119^, ^120^, ^121^, ^122^, ^123^, ^124^, ^125^, ^126^, ^127^, ^128^, ^129^, ^130^, ^131^, ^132^, ^133^, ^134^, ^135^, ^136^, ^137^, ^138^, ^139^, ^140^, ^141^, ^142^, ^143^, ^144^, ^145^, ^146^, ^147^, ^148^, ^149^, ^150^, ^151^

### Key‐Informant Interviews

The study received institutional ethics approval from the London School of Economics and Political Science (reference number 245167) and was deemed low risk given the focus on public policies. Key informants were identified through document analysis and snowball sampling. Individuals were contacted via email. At the end of each interview, participants were asked for suggestions for others to include in the study. A total of 36 individuals were contacted, and seven declined to participate (one participant explicitly declined; the other six did not answer follow‐up emails to participate). The use of the data, confidentiality, and ability to withdraw at any time were discussed during the consent process, along with the researcher background, study purpose, and dissemination plan.

A total of 29 individuals participated in the semistructured interviews (81% acceptance rate). Fourteen were from international organizations (e.g., WHO), six from NGOs, five from pharmaceutical companies, and four clinicians who had provided technical advice for recommendations (categorized under “international organizations” given their role in this research). These broad categories are used (as opposed to specific organizations or companies) to encourage openness and promise anonymity to the participants. This was necessary given the nicheness of the topic area. Each participant had direct experience in the selection of essential medicines at the WHO, either as former Expert Committee members, in technical departments, as applicants, or in observing organizations or companies. Each participant spoke from their own experience and was not representing views on behalf of their affiliated organization. Participants were from seven countries (Switzerland, Italy, England, the United States, the Netherlands, Canada, and South Africa). Given the focus on global health governance, nearly half the sample (15 of 36; 42%) were affiliated with international organizations in Switzerland. However, nearly all participants had experience working in LMICs.

Elite interviews suffer from common limitations, such as misrepresentation, omission, or recall bias.^152^ Therefore, a broad range of actors was purposively sampled to triangulate responses from the interviews and document review. Participants were asked open‐ended questions relating to the structure, process, and outcomes of the WHO EML. This included significant events related to essential medicines over the past two decades, how “essentiality” is understood, the challenges and opportunities of the WHO EML, and the WHO policy environment. Political factors (ideas, interests, and institutions) were used to probe narratives and elicit further information. This approach allowed for comparability among interviews even when the subject matter differed. Three interviews were repeated to probe further about specific topics deemed important by several participants (e.g., the 2015 update in which several novel medicines were added, the challenges posed by cancer medicines). The interview guide was not pilot tested, but questions were refined throughout the study. An example of the questions that were asked is included in Appendix 4. The interviews lasted approximately 1 hour and were conducted online (and audio recorded) via Zoom or Microsoft Teams. Transcripts were not returned to participants for further comment. Because of the highly specialized nature of the research field and the assurance of anonymity provided to participants, the interview transcripts will not be shared publicly.

### Data Analysis

Key‐informant interviews were anonymized and transcribed using Otter AI. The interview and document review data were analyzed using deductive and inductive thematic analysis using the NVivo software package (version 12.7.0).^12^ The concepts of ideas, interests, and institutions were used as initial deductive codes. Other inductive themes were recorded as data analysis progressed. In line with thematic analysis, the document review, interviews, and analysis continued iteratively until data saturation. The Consolidated Criteria for Reporting Qualitative Research (COREQ) guidelines were used to ensure accurate reporting of the methodology and results.^13^ The COREQ checklist is included in Appendix 5.

## Results

**Figure 2:**
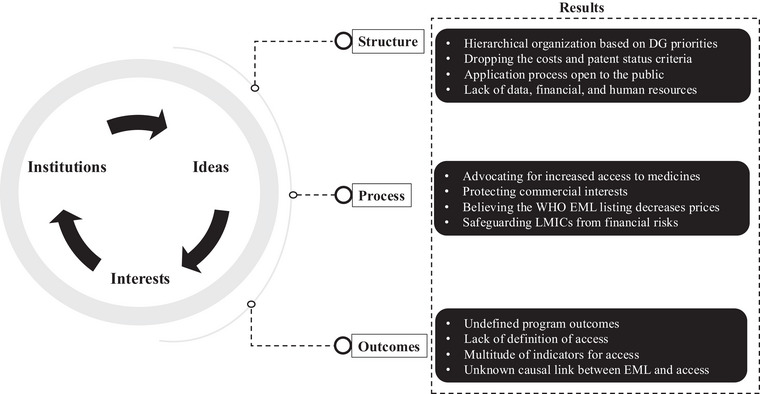
Merged Theoretical Frameworks With Major Themes From the Document Review and Key‐Informant Interviews DG, director‐general; EML, Model Lists of Essential Medicines; LMIC, low‐ and middle‐income country; WHO, World Health Organization.

The results are presented in the domains of the Donabedian model for quality, starting with a background on the evolution of the WHO EML processes, moving to how ideas and interests intersect with institutional factors in defining the program's structure, the process to select medicines, and the definitions of outcomes. An overview of the major themes identified through the document review and key‐informant interviews mapped onto the merged theoretical frameworks are provided in Figure 2.

### Background on the Evolution of the WHO EML

The WHO is the UN specialized agency responsible for international public health. Within the WHO, the Department of Health Products and Policy Standards oversees the development and maintenance of the EML. This department, situated under the broader umbrella of the Access to Medicines and Health Products epartme, is responsible for the prequalification program, maintenance of the international pharmacopoeia, the Model Lists, and trilateral cooperation on intellectual property protection among other items.

**Figure 3:**
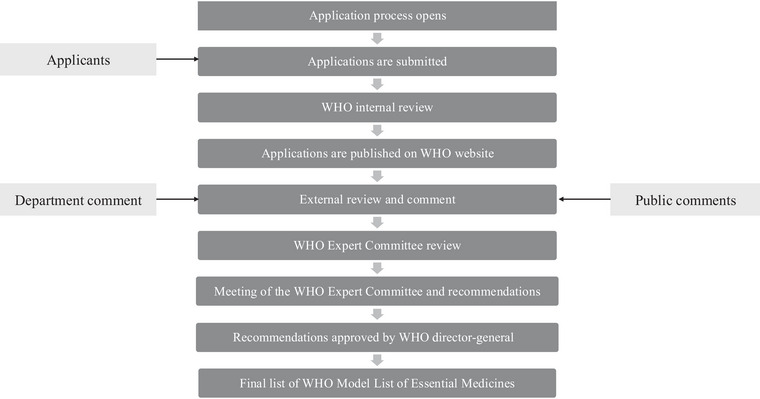
The Process for Updating the WHO Model Lists of Essential Medicines WHO, World Health Organization.

The WHO EML is updated every 2 years through an application process that opens approximately a year before the update. Applications are submitted and internally reviewed before being published on the WHO website. Once the applications are publicly available, external stakeholders including technical departments, applicants, and other members of the public can submit comments to the secretariat. These comments are made publicly available on the WHO website. Applications and external comments are reviewed by the WHO Expert Committee on the Selection and Use of Essential Medicines (Expert Committee)—a committee of experts contracted to make nonbinding recommendations that are subject to final approval by the WHO director‐general (DG). An overview of the process of updating medicines listed on the WHO EML and relevant stakeholders is provided in Figure 3.

In 2001, the WHO revised the definition of an essential medicine and decision criteria, marking the first time changes were made to the procedures since its inception in 1977.^153^ The original definition of essential medicines from 1977 was expanded into an evidence‐based process,^153^ with specifications that each application should consist of data supporting the clinical benefit, safety, and comparative cost‐effectiveness. The Expert Committee meeting was privatized with strict procedures for managing and reporting conflicts of interest. The pharmaceutical industry and patient advocacy groups could no longer participate as observers; instead, an “open session” was introduced in 2005, during which public members could comment on matters related to the meeting.

The 2001 Revised Procedures introduced two fundamental changes to the decision‐making process that may have had unintended consequences for the secretariat given the evolution of the pharmaceutical ecosystem. First, the “total costs” and “patent status” criteria were removed as decision criteria. Before 2001, costs were a “major consideration” for a medicine's inclusion in the WHO EML. Therefore, after 2001, affordability was no longer a central feature in deciding whether a medicine could be essential. As one participant explains, “The key point is that affordability, which used to be a selection criterion, became a result of the selection” (participant 1). This change facilitated the addition of 12 antiretroviral drugs to the WHO EML, marking the first time high‐priced medicines were deemed essential.^2^ Although there are secondary criteria, such as affordability and feasibility factors, the 2001 Revisions intentionally weighed clinical benefit. The idea was that cost is variable across countries; therefore, the WHO would guide countries clinically.

Dropping the costs and patent status criteria allowed for the addition of several complex, high‐priced medicines over the years, shifting the concept of essential medicines from its original focus on LMICs (the result of strong pharmaceutical lobby in the first years of the program^21^, ^22^). The WHO emphasized the Model Lists as a “global standard”^154^ and “equally relevant for [high‐, middle‐, and low‐income] countries”^4^—language that was echoed by members of the global health community who published articles extrapolating the essential medicines concept to high‐income countries,^7^, ^155^ including the United States.^6^

The second substantial change to the structure of the WHO EML was introducing a public application process whereby any individual could apply for a medicine to be essential. Originally, additions to the WHO EML were the result of the pharmaceutical industry and technical departments.^2^ Although the essential medicines concept may have been reactive to these stakeholders before the 2001 revisions, the open application process meant that a more diverse range of individuals could shape the concept of essential medicines through applications. A wide range of actors (e.g., international organizations, UN bodies and WHO technical departments, universities, professional societies, and pharmaceutical companies) began submitting applications for medicines that were traditionally not considered essential, such as high‐priced medicines for cancer and rare diseases.^25^ These medicines challenged the original definition of an essential medicine given small patient populations and frequent requirements for complex administration.

### The Structure of the WHO EMLs

**Figure 4:**
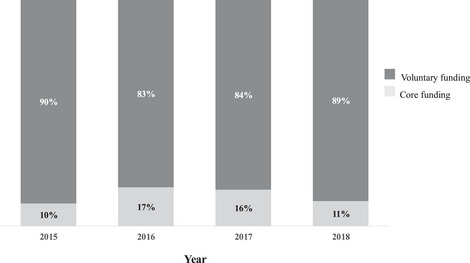
Overall Distribution of Voluntary and Core Departmental Funding Data were sourced from the departmental annual reports. The years (2015‐2018) were chosen because of the public availability of these reports. Funds are not adjusted for inflation. Data are not available per subdepartment.

The power of applicants to shape the Model Lists is exacerbated by a lack of funding and personnel to conduct systematic evaluations of the WHO EML to ensure there are no missing medicines. The WHO has stated that the application process suffers with the “ad hoc and volunteer basis, with insufficient resources and enforcement powers to oversee and rebalance selection and other bias inherent in a volunteer‐based process by applicants with conflicts of interests.”^156^ To my knowledge, WHO budgetary allocations are not available for activities within a department (e.g., between clusters). However, over the past 20 years, research has demonstrated a decline in assessed contributions to WHO core budget and overall operations.^157^ Simultaneously, there has been a rise in the voluntary contributions earmarked for specific activities chosen by donors.^157^ Between 2015 and 2018, voluntary contributions accounted for nearly 90% of the department's budget (Figure 4).^158^, ^159^, ^160^, ^161^ However, this was not always the case. In the first 25 years, the program had financial support and personnel dedicated to advancing the concept of essential medicines. However, funds were mostly allocated to the regulatory function of the department. Participant 28 (international organization) said, “Well, you can see a shift over time, and most of the funding is currently on the prequalification program. I think it's diversifying a bit now, but in the early days in the eighties and the nineties, there was much more funding for the essential medicines concept.”

Regarding human resources, at the time of this research, the WHO had two full‐time staff members and a handful of consultants to maintain activities related to essential medicines. This includes various activities such as updating the Model Lists, supporting countries in their national formularies, consulting with applicants, reviewing applications, and formulating policies to encourage appropriate use of medicines among other administrative activities within the department. A lack of staff raised the following question from participant 28 (international organization) about sustainability: “A big question is how sustainable is all that? Because WHO is running a huge, scientifically complicated operation with two or three people.”

Staffing issues are not unique to the department but are widespread at the WHO. In 2020, the WHO established the COVID‐19 technology access pool, which was run part‐time by one individual because of funding restrictions (participant 12, NGO). Furthermore, technical departments do not exist for all diseases, leaving some areas without political priority, therefore accentuating a potential gap on the WHO EML.^162^ The lack of guidelines and WHO guidance on certain areas challenges the secretariat given there is no guidance on which treatment regimens ought to be prioritized:

I think WHO also really failed to prioritize and identify gaps in the EML for very common diseases, very prevalent in LMICs, but I wouldn't say it's specific to the essential medicines area. I think there should be WHO guidelines for sickle cell disease. There's nobody who takes these on. I believe in the whole [noncommunicable disease] area; even if you go beyond cancer, if you talk about rheumatologic diseases, skin diseases, autoimmune diseases, it's been very weak.

—Participant 26 (international organization)

In sum, the change in decision criteria to drop the costs and patent status criterion may have introduced unintended consequences given the evolution of the pharmaceutical industry toward complex, high‐priced medicines for which prices are hard to ignore. The open application procedures, although equitable, introduced a process in which a diverse range of stakeholders could shape the Model Lists given their interpretation of what constitutes essential. Given a lack of human and financial resources, there are no mechanisms to rebalance selection if applicant priorities do not align with the WHO.

### The Process of Selecting Essential Medicines

The selection of essential medicines involves diverse stakeholders, each with their interests and ideas as to what constitutes an essential medicine. This section is divided in two. First, interests of various stakeholders are outlined sequentially in the stages of the selection procedures given these are the major actors that shape the process (Figure 1). Second, conflicting ideas about the role of the WHO EML in promoting access to medicines are outlined. The different views presented in this section demonstrate a clear lack of consensus on the role and purpose of the WHO EML. As inputs into the decision, along with scientific evidence, conflicting ideas and interests add further uncertainties into the process to select essential medicines.

*Interests*. Participants who submitted applications described how an “essential” designation could emphasize a medicine's “exceptional status,” therefore increasing access through a variety of measures. An example of this type of advocacy can be found in a Multiple Sclerosis International Federation webinar titled, “Key Steps to Get Started in Using the WHO EML in Your Advocacy Efforts,” conducted shortly after their 2023 application received a positive recommendation.^163^ The webinar provides individuals and NGOs within the multiple sclerosis community with guidance for how to influence decisions through engagement with policymakers.

Applicants also described scenarios in which an “essential” designation may help governments pursue access through other means, such as compulsory licenses. However, the opposite is also true. As one participant explained, exclusion from being essential could weaken a medicine's “exceptional status,” making it harder for a country to use the Trade‐Related Aspects of Intellectual Property Rights (TRIPS) flexibilities. Participant 9 (NGO) said, “If [countries] apply for a compulsory license and a drug is not on the essential drugs list, then the companies say you should not. Or the US Trade Representative says, don't issue a compulsory license. That's not even on the essential drugs list.”

WHO technical departments and working groups support the WHO EML by reviewing supporting evidence for medicines, providing public comments, and submitting applications. Although technical working groups are ubiquitous at the WHO, there are two groups that were formed to support the secretariat with antibiotics and cancer medicines. As with all individuals, these groups have interests and policy goals that are not always aligned with the WHO EML. For example, the noncommunicable disease department and cancer medicine working group have hesitated to recommend cancer medicines because of the high prices, even when this advice contradicts official criteria for cancer medicines.^11^ When asked about this challenge, one participant described how excluding a drug from the WHO EML would take a stand against the pharmaceutical industry (participant 14, international organization). In other words, there seems to be an advocative strategy to signal to the pharmaceutical industry that they must lower prices before becoming essential. Given the low proportion of applications from industry, the impact of this advocacy strategy is unknown.

The pharmaceutical industry submits the lowest proportion of applications to the WHO EML,^25^ suggesting a lack of interest or active skepticism about the program. However, industry engages in different ways. For example, when other stakeholders submit applications for patent‐protected products, industry protects their interests. The International Federation of Pharmaceutical Manufacturers Association (IFPMA) routinely submits public comments and attends the open session during each EML update. The IFPMA has raised concerns about the WHO EML's role into “market‐shaping activities,” exclusion from the technical working group, role and function of the Model Lists, and decision‐making transparency.^164^

The pharmaceutical industry is a heterogenous group of actors.^165^ Some companies are sensitive to access issues and have long legacies of philanthropic activities. For some, the Model Lists can be used to advance corporate social responsibility goals “as long as it does not create a loss for the company” (participant 5, industry). One industry participant explained the complex relationship between industry and the WHO EML:

So many factors are playing a role here. I think a lot of it also has to do with personal views on [the] EML. If some experts working on the company see this as an important political tool that might play a role in whether it's in a positive lens […] However, there might be more pressures for price reduction, reimbursement, etc. That will depend on the person you have leading the portfolio

—Participant 22 (industry)

The pharmaceutical industry is particularly sensitive to the potential impact of the WHO EML on medicine prices (participants 5 and 29, industry). In 2018, the WHO established the Essential Diagnostics List (EDL), using similar principles of rationalization as the EML. Fears that the EDL would lead to price capping was mentioned by 40% of industry representatives at the 2018 McGill Summer Institute in Infectious Diseases and Global Health.^132^ Fears about impacts on markets and the EML were made explicit in a letter from two US diagnostic lobby groups to Suzanne Hill, the former Director of the Health Products and Policy Standards. They state the following:

Taking lessons learned from the implementation of the Essential Medicines List (EML) of many decades, we understand that member states have utilized the EML to implement restrictive pricing of medicines to drive down their cost. The implementation of price controls on diagnostic tests… could lead to stifled innovation and reduced access to diagnostic tests… The true value of diagnostic tests goes well beyond price.^112^

Industry participants acknowledged the system they must work within (i.e., “answering to shareholders”). Therefore, any “market‐shaping activities” will be monitored:

The IFPMA started watching the EML more closely when WHO started listing patented drugs. So, at the beginning of the first EML update in 2015, [WHO] was brought to the World Intellectual Property Organization. They wanted to know how many patented drugs are on the EML and how many should be. Will the number increase?

—Participant 21 (international organization).

When recommendations do not align with commercial interests, one industry participant described national advocacy strategies to align countries to their perspectives:

Whenever we see a decision being taken at a global level that is not playing in our favor, then what we try to do is look at the national context and make sure that we can do some national advocacy to make sure that countries understand what were the processes behind any international decision making that led to this outcome and give them our views on how that should be applied to the national level.

—Participant 22 (industry)

Given the power to select and recommend medicines for the WHO EML, the Expert Committee exerts considerable influence on the Model Lists. Expert Committee members are ultimately appointed by member states, introducing both strengths and challenges to its structure. On the one hand, this approach ensures direct accountability to WHO member states, aligning decisions with global health priorities. On the other hand, it may introduce political considerations into the appointment process, potentially influencing the committee's composition and decisions. One participant recounted an instance in which members expressed feelings of exposure during recommendations given their perceived accountability to their governments (participant 7, international organization). Furthermore, the infrequency of meetings (once every 2 years) can delay the timely consideration of emerging health needs.

Lastly, the DG is responsible for approving collective recommendations by the Expert Committee for including, changing, or deleting medicines on the Model Lists. Although it is rare that the DG would alter a recommendation, modifications have happened. For example, in 2005, the inclusion of mifepristone with misoprostol for medical abortion was altered with an asterisk, indicating that the recommendation applies specifically to countries where abortion is legal.^166^ The DG is a political position that must balance WHO outputs, primarily technical guidance, with the interests of member states and donors. This is especially acute in pharmaceuticals, in which powerful countries (e.g., the United States, Germany, Switzerland) lobby often against essential medicines policies that may negatively impact commercial interests within their jurisdictions (participant 12, NGO).

*Ideas*. In 2003, Pierre Chirac, a representative of Médecins Sans Frontières (MSF), writing about the essential medicines concept, theorized that the WHO EML had three functions: 1) a tool to enable policymakers to identify drugs for health care systems, 2) a reference for practitioners to identify drugs that ought to be prescribed given crowded markets, and 3) a mechanism to signal to the world that a medicine must be made available—a term he called the “symbolic function” of the Model Lists.^167^ Although the first two functions are relatively straightforward, the “symbolic function” is less obvious. Chirac provides an example of the World Trade Organization meeting in Seattle 1999 at which the European Commission proposed to exclude essential medicines from patents.^167^ Although there is no formal relationship between trade agreements and the WHO EML, this example demonstrates an ideological link between the concept of “essentiality” and real‐world political advocacy efforts that may increase access, underscoring a potential power the Model Lists may have.

The belief that WHO EML listing may increase access is nearly always discussed through its impact on lowering prices. For example, a recent editorial outlining concerns about the inclusion of medicines for bleeding disorders stated that “the inclusion of medicines to the Essential Medicines List results in pressure to decrease costs, as shown with medications for diabetes.”^15^ However, the referenced article to this statement did not provide evidence for the impact of the Model Lists on prices. Rather, it estimated price targets that governments could pursue in negotiations on NEMLs given the addition of several medicines for diabetes to the Model Lists.^168^ In fact, their results also demonstrated large incremental price increases for insulin glargine after their addition to the WHO EML.

Despite conflicting evidence for the impact of the Model Lists on prices, this ideology is widespread throughout WHO policy documents and peer‐reviewed literature. The emergence of this idea can be traced to the 2001 Revised Procedures, which stipulated that affordability was no longer a precondition for listing. Although there was discourse about the Model Lists and prices before the 2001 reforms, the introduction of several highly effective, yet expensive, medicines in the early to mid‐2000s increased the prevalence of this idea given uncertainty about the impact of listing. Without any data, this idea that the WHO EML would decrease prices was never evaluated and continued to underpin recommendations (Table 1). Participants described this discourse as “the HIV narrative”—a belief that WHO EML listing facilitated price decreases for antiretroviral drugs after they were added to the Model Lists in 2002. An example of “the HIV narrative” is exemplified in a study that interviewed WHO staff who stated, “In 2002 […] there were about 30 [antiretroviral drugs] on the market, for the EML we chose 12 and they were very cleverly chosen. [….] Three years later, 85% of all medications available were these 12. It basically knocked out the other 18 and drove prices down.”^20^

**Table 1:** Other Representative Quotes per Framework Domain

Domain	Quotes
Structure	“This department has always been somehow focused on the work on essential medicines, has gone through a number of cycles [of] becoming large and being able to do [the work of the] EML. Then it also has periods where funds are more limited.” (participant 4, international organization)
	“The EML has suffered from being completely understaffed compared to the task at hand…most of the staff won't evolve within a biennium […] no long‐term planning is possible.” (participant 26, international organization)
	“People are always complaining about the WHO but then do not give the resources that are necessary to make it work. And that remains a huge problem.” (participant 12, nongovernmental organization)
	“The key point is that affordability became the result of listing.” (participant 1, international organization)
Process	“No more industry there [expert committees], no more patients’ groups. Instead, [WHO] made an open session. So, the first half day of the week meeting is an open session where everybody can come and if they want to lobby and present, they can do that in public.” (participant 1, international organization)
	“The HIV narrative…. we can no longer say we can't do this because it's too expensive. Because if the HIV people had done that, we wouldn't have HIV medicines. And so, my thinking about this is that while there's a lot that we can learn from the HIV scenario and they accomplished huge things, it is so different as the treatment for HIV is obviously much simpler [compared with cancer], but it's also so incredibly effective and it takes a terminal disease and turns it into a disease where people live for decades.” (participant 14, international organization)
	[On process and outcomes] “There's truly a lack of understanding of how the EML not only functions in terms of process…but also what it means once the product is on the EML, what does that trigger concretely for patients?” (participant 22, industry)
Outcomes	“I think the antibiotics [were] important. So, I think in that respect, strengthening the methodological part of it, which particularly [specific members of the secretariat] have been doing, you can see that over time from a methodology point of view, that has greatly improved.” (participant 26, international organization)
	“The natural question we have is what impact does the existence of a WHO model list have on access to medicines and use of medicines? And that sort of question can be answered by looking at data […] These sorts of questions are important for an organization like the WHO to ask and to have answers to as it pursues the policy of updating and promoting the notion of the model essential medicines list and encourages health systems around the world to also have their essential medicines list for which the WHO provides a model.” (participant 27, industry)

EML, Model Lists of Essential Medicines; WHO, World Health Organization.

In 2013, the symbolic function of the Model Lists was codified into decision criteria when the public health relevance criterion was redefined to include “the potential political impact of identifying a medicine as essential for advocacy purposes.”^169^ At the same time, the WHO was struggling with adding several high‐priced, yet effective cancer medicines to the Model Lists (e.g., trastuzumab for breast cancer, imatinib for leukemia), which resulted in a comprehensive review of the cancer section.^170^ Again, the HIV crisis and the addition of antiretroviral drugs were cited as reasons the WHO believed “adding a medicine to the WHO EML might precipitate a ‘special intervention’ before the normal processes of patent expiry and could be used as an advocacy tool to reduce the price of medicines.”^169^

In 2015, several complex, high‐priced patented medicines were added to the WHO EML for cancer and other therapeutic areas (e.g., hepatitis C).^4^ These additions marked the second time since 2002 that several patented medicines were designated as essential, shifting the definition of an essential medicine.^8^ Given that their prices were unaffordable at the time of listing, the WHO repeatedly emphasized the symbolic function of the Model Lists. In an editorial accompanying the publication of the 2015 recommendations, authors from the WHO secretariat stated that the inclusion of novel medicines despite high prices is “expected to support efforts to reduce the prices.”^4^ Since 2013, the bidirectionality between prices and EML listing has been acknowledged explicitly in Expert Committee recommendations. For example, in 2023, the Expert Committee excluded several immune checkpoint inhibitors primarily because of price; however, they considered “that the availability of several immune checkpoint inhibitors as therapeutic options can boost competition and facilitate affordable access.”^171^ This discourse was also reflected among prominent NGOs, such as MSF, who in response to the 2015 additions of high‐priced medicines, stated that “having these medicines on the EML can precisely create the pressure that is needed to overcome pricing and access barriers.”^172^

Scholars and participants within the study noted how the symbolic function of the Model Lists is often applied inconsistently across therapeutic areas. In 2023, Hwang and colleagues noted how some high‐priced therapeutics (e.g., diabetes or hepatitis C) receive positive recommendations, whereas cancer medicines within similar complex status and prices do not.^11^ When asked about these discrepancies, several participants noted additional complexities with cancer medicines. For example, the safety and delivery of cancer medicines in LMICs is not established. Given the need for trained staff and specialized facilities, cancer medicines require more feasibility considerations. However, it is also the case that cancer medicines have their own working group (along with antibiotics) that support the WHO EML through reviewing evidence and providing input into recommendations for which medicines ought to be included on the Model Lists. Costs are a major factor for this group, as evidenced by their recent advice for the 2023 update. They state, “The group did not reach consensus regarding the addition of pembrolizumab and other immune checkpoint inhibitors for non‐small cell lung cancer (NSCLC) to the Model Lists. While the inclusion could be supported on their clinical efficacy and safety, there are major concerns about financial risks.”^12^

The belief of the symbolic function of the Model Lists determines whether participants deem complex, high‐priced medicines should be included in the WHO EML or whether they should not. For example, those that believe that the WHO EML is a tool to facilitate lower prices and increased access advocated for inclusion of complex medicines (e.g., monoclonal antibodies, cell and gene therapies, or immunotherapies). These individuals underscore the spirit of the 2001 Revisions, which state that affordability should be the result of the WHO EML not a precondition. However, if participants do not believe the Model Lists have these powers, they advocated against including complex, high‐priced medicines. One participant described the risks of placing high‐priced medicines on the WHO EML given the “opportunity costs” that may divert resources away from other areas (participant 14, international organization). As such, a divide has emerged between those who believe the symbolic function can increase access (i.e., lower prices) and those who believe it cannot.

The interpretation of the WHO EML's role in global priority setting is also closely tied with the individual's belief in the symbolic function of the Model Lists. Such as the case with complex, high‐priced medicines, if an individual believed these medicines should be included given the favorable impact on prices and access, then they also believed that the Model Lists were relevant to high‐income countries. Similarly, the opposite was true. Participant 1 (international organization) said the following: “The Model Lists are a model for middle‐income countries now struggling with establishing universal health coverage through social health insurance. They are the ones that need help. And that's where the model works.”

In sum, there are several actors that shape the process of selecting medicines for the Model Lists. These individuals are applicants, technical advisers, WHO staff and leadership, member states, Expert Committee members, and the public (e.g., in the open session)—all with varying interests and resources as advocates or critics. In 2013, an idea about the WHO EML's symbolic function was strengthened given the increase of highly effective yet high‐priced medicines and again when it was codified into decision criteria. The current debate for whether the Model Lists should include complex, high‐priced medicines depends on the individual's belief whether the WHO EML can facilitate access (i.e., price decreases) after listing. Those who believed that the WHO EML had no impact on access (i.e., prices) advocated strongly against inclusion of these medicines and tended to favor a focused remit on LMICs.

### The Outcomes of the WHO EML

Donabedian stated that “outcomes, by and large, remain the ultimate validators of the effectiveness and quality of medical care.”^26^ When applied to the WHO EML, a logical question arises: Which outcomes matter?

A natural answer is improved access to essential medicines. Indeed, participants within this study agreed that the WHO EML has a major role in improving access to medicines and universal health coverage. Even participants from industry, while working within the constraints of a for‐profit business model, recognized the importance of the WHO EML in global health advocacy efforts. However, there was no agreement on the mechanisms that facilitated improved access or which indicators mattered most. A major focus of the first 25 years of essential medicines was on improving the rational use of medicines.^173^ While this remains a central aspect of the program's ethos, additional metrics have been introduced over the years, potentially blurring outcomes. For example, in 2003, in response to the World Health Assembly resolution 54.11, the first edition of the WHO/Health Action International (HAI) survey was published. The main objective of the survey was to generate data on the price, availability, and affordability of essential medicines within countries.^174^ The WHO/HAI methodology forms the basis of the access to medicines indicator in the Millennium Development Goals and Sustainable Development Goals. In addition to rational use, “access” now included dimensions of affordability, availability, and price. In 2017, a database of 137 NEMLs was published by academics in collaboration with the WHO.^175^ This database has generated a large subcategory of literature comparing the proportion of medicines listed on NEMLs with those on the WHO EML,^92^, ^176^, ^177^ ^‐^ including across high‐income countries.^178^ Now, “access” also included the proportion of WHO‐designated essential medicines on a country NEML.^176^ Utilization, prices, affordability, availability, and NEML comparisons are often used interchangeably as access indicators with limited appreciation of the causal pathway from WHO EML listing. The following question remains: Which outcomes matter?

Data are critical to conducting program evaluation to understand the impact of the WHO EML on global utilization trends, sales, and prices. However, these data do not exist for the WHO. The HAI/WHO surveys form the basis of the access to medicine indicator (3.b.3) of the Sustainable Development Goals. This indicator has always suffered from low reporting, even during the Millenium Development Goals.^140^ Currently, the WHO database includes only 24 surveys, most of which are outdated.^179^ Participant 7 (international organization) said, “WHO [has] almost no data apart from HAI WHO surveys on affordability and a few availability surveys in individual countries, but there isn't a well‐entrenched methodology for measuring either access or affordability of medicines bizarrely.”

Commercial data are notoriously sparse outside of high‐income country markets. Data companies must invest substantial resources to collect accurate sales and volume data within countries. The pharmaceutical industry shapes which countries’ companies invest resources to collect these data as the primary consumers of these data:

Data is a commercial business, so we must be able to make money, and we spend a lot of money gathering the data. We need customers who will find value in our data, which is typically led by pharmaceutical manufacturers. However, many of them don't have a direct presence in low‐income countries. And frankly, their level of interest in the market is very low.

—Participant 27 (industry)

There are two notable examples in which the WHO attempted to use commercial data to evaluate the impact of the WHO EML. In 2015, the WHO wanted to assess the effect of EML listing on sales and volumes worldwide. However, the findings were never published because of a lack of agreement between the WHO and the commercial data company regarding specifics in publishing the report. At the time, the WHO legal department had reservations about the use of commercial data for the country analyses. Simultaneously, commercial data companies have strict licensing agreements that limit data sharing, even within the same organization. There was no middle ground, and the report was never published. Participant 21 (international organization) said, “WHO had data showing the percentage of essential medicines covered in various countries. There were roughly a hundred countries that we were almost ready to prepare or try to publish. But the [WHO] legal department was against it because the data was from a for‐profit organization.”

The second time, the WHO partnered with external academic partners to evaluate worldwide utilization of antibiotics.^88^, ^180^, ^181^ From these data, the WHO developed the Access, Watch, Reserve (AWaRe) classification system and an antibiotic guidebook for treating common clinical infections.^143^ Several participants mentioned the positive impact of the activities for antibiotics. However, antimicrobial resistance has received higher political priority in recent years, which likely underpins this success unlike other therapeutic categories like cancer. For the WHO, political will stems from member states within the World Health Assembly and the executive board and, most importantly, manifests as funding to undertake certain activities (participant 12, NGO; participant 28, international organization). For the AWaRe book, the WHO received voluntary funding from several donors, including high‐income country member states, such as the United Kingdom (participant 26, international organization). Rational use was cited as an outcome of this initiative.^182^ However, it is also the case that the proportion of essential antibiotics on NEMLs has also been used as an indicator for successful AWaRe implementation too, underscoring the various indicators used by different stakeholders.^88^, ^183^

In sum, outcomes are a critical part to measuring health care quality or, in this case, the impact of the WHO EML. However, when the Model Lists are applied, there is no clarity on which outcomes matter and for whom, complicating future evaluation and adding uncertainties in recommendations given a lack of understanding on the effect of listing drugs on the Model Lists. The lack of WHO data and challenging access to commercial data mean there is limited evaluation of the impact of the Model Lists.

## Discussion

Over the past 50 years, several actors and their interests and ideas have shaped the structure, process, and outcomes of the WHO EML.^21^, ^22^ The concept of essential medicines evolved from an original focus on generic medicines in resource‐constrained countries to include complex, high‐priced therapeutics relevant to high‐income settings. The WHO has never explicitly addressed whom decisions are for, leaving the interpretation of an essential medicines to different perceptions. Given the multitude of stakeholders involved in shaping the WHO EML, this has led to different ideas about which medicines should be included and their impact driving the Model Lists. A lack of data exacerbates uncertainties in decision making given increased applications for complex, high‐priced medicines and their unknown consequences in resource‐constrained countries. As such, a question about the purpose of the WHO EML has emerged in recent years, dividing stakeholders on the mandate and future vision of the Model Lists.

The current debate centers on the question of whether the WHO should include complex, high‐priced medicines on the EML.^10^ However, this research demonstrates that present challenges have roots much deeper than solely amending listing criteria or adopting evidence‐based decision frameworks. At the core of this issue is the purpose of the Model Lists. The pharmaceutical landscape has changed substantially over the past 20 years. Participants were divided on the definition of an essential medicine, with some stating that the Model Lists should include complex medicines and high‐income countries and others advocating against. A problem occurs because these participants all had inputs into WHO EML decision making. The outcome is a clash of ideas and interests with very little institutional structures to align stakeholders.

International organizations are not politically neutral sites but arenas in which diverse individuals struggle to shape outcomes through formal and informal means.^184^ Internal technical groups have lobbied against adding complex, high‐priced medicines to protect LMICs from financial strain. Simultaneously, some individuals believe that including these medicines as essential may decrease prices and increase access. Powerful actors with substantial financial resources and access to data, such as pharmaceutical companies and their governments, lobby against the broadening role of essential medicines. Assessed contributions, which fund WHO core activities like the Model Lists, have decreased over the past 20 years and weakened the organization's ability to conduct evaluation and strategic planning, exacerbating the influence of external actors. As a result, a degree of inconsistency has emerged, both in the concept and the recommendations for essential medicines.

Contemporary challenges with complex, high‐priced medicines has led the WHO to recently announce a process to revise the selection procedures for the EML.^156^ Authors close to WHO EML processes recently suggested ten visions for improvement, centered on building transparency, strengthening dissemination to countries, and improving affordability.^185^ This research aligns with several of these recommendations and underscores the importance of articulating the role and purpose of the WHO EML to align diverse stakeholders toward a common goal and reduce inconsistencies in recommendations. However, it is also important to recognize that ambiguity can also serve as a political strategy, offering flexibility in interpretation, facilitating the accommodation of competing interests, and helping to maintain consensus among stakeholders with differing priorities. This is especially relevant in contentious areas such as pharmaceuticals, for which the WHO EML sits at the intersection of competing interests. Balancing the call for greater clarity with the need for flexibility and consensus is important, as overly defining the WHO EML's role could risk reducing adaptability, heightening political tensions, and undermining the broad stakeholder support that sustains its legitimacy.

There is a current question whether the WHO should consider costs when recommending essential medicines. The 2001 Revised Procedures stipulate that neither costs nor patent status can be reasons for a medicine's exclusion from the WHO EML. However, these criteria were predicated on a one‐off issue (HIV/AIDS), and the WHO did not foresee the future challenges of not considering cost as a key criterion. Research and development have trended toward medicines for rare diseases and cancer, many offering marginal improvements over existing therapies.^186^ It may be difficult not to consider costs with marginal clinical benefits. Interestingly, when asked about their definition of an essential medicines, all participants noted that essentiality pertains to their clinical benefit, not costs. However, confusion ensued for how this translates into decision making for medicines on the Model Lists given a lack of consensus of the program's purpose and outcomes. Here, evidential frameworks, such as GRADE, will greatly improve consistency within decision making.^19^, ^185^ Currently, these tools are not used systematically in decision making.^156^

The WHO must have adequate structural resources to realize its strategic vision, including sufficient financial and human resources and data. Without these supports, the future impact of essential medicines is uncertain. Encouragingly, participants mentioned ongoing analyses at the WHO of the impact of the Model Lists on prices using commercial data, suggesting some lenience in the institutional barriers of 2015. However, according to one participant, these analyses only include a few medicines. If this is the case, then more comprehensive analyses are needed, especially in areas that dominate applications and challenge recommendations, such as cancer.^187^ Member states ought to support the WHO with resources (data and personnel) to conduct evaluation and undergo reforms on the selection procedures.

### Limitations

This research must be interpreted with several limitations. First, qualitative research is inherently interpretative and shaped by the researcher's engagement with the data. This paper is a personal balancing act between being an academic observer of the WHO and a former consultant supporting the Model Lists. At the WHO, I gained an appreciation for the complexity of global health governance and the myriad of actors at the international level, each with their own power and interests that shape what can and cannot be done. This research was motivated by a desire to understand these influences and provide additional context into the current debate on the future of the Model Lists. However, being too close to an organization when undertaking research runs the risk that one feels they cannot address criticisms. On the other hand, personal experience can also enable a rich understanding of the inner workings and lead to better characterizations of the challenges and opportunities. To address unnecessary bias, data were triangulated with several written sources and a wide range of stakeholders that intersect with the WHO EML were interviewed, including critics. However, a degree of subjectivity remains, and the results must be interpreted with the position presented here.

### Conclusion

This analysis investigated the perspective of interests, ideas, and institutions to explore the political factors that influence the structure, process, and outcomes of the WHO EML. Since 1977, the concept of essential medicines has evolved substantially. Once a concept for resource‐constrained nations, essential medicines were redefined to include several complex, high‐priced medicines widely used as front‐line treatment in high‐income countries. As such, the definition of essential has shifted over the years, influenced by diverse stakeholders with different ideas and interests. A lack of structural support, in terms of adequate data and financial and human resources, inhibits evaluation and perpetuates inconsistencies in recommendations. Solutions to current challenges have focused on ameliorating technical aspects of the WHO EML, such as implementing evidence‐to‐decision frameworks, improving the connection to WHO guidelines, or implementing separate processes to evaluate economic and clinical evidence.^10^, ^11^, ^19^, ^20^ Although technical aspects are important, these solutions do not consider the political context of the WHO. Conflicting ideas about the Model Lists’ impact on prices, a lack of data and resources to conduct evaluation within an ecosystem that includes a well‐resourced pharmaceutical industry and their governments that lobby to protect commercial interests, and a lack of consensus on the purpose and outcomes of the WHO EML will severely constrain what can be done. This research underscores the importance of strong institutional structures that can be generalized to other major organizations. Defining a strategic vision for the WHO EML, refining decision criteria, and adequate structural support would align interests toward a common goal, supporting good processes and, ultimately, contributing to positive societal health outcomes.

## Funding/Support

K.J. was supported by a Canadian Institutes of Health Research Doctoral Foreign Study Award (181603).

## Conflict of Interest Disclosures

No disclosures were reported.

## Appendix 1

Study Design

## Appendix 2

Key Organizations Consulted During the Targeted Literature Search

**Table:**

Organization Type	Name	Link to website
Civil Society & NGOs	Knowledge Ecology International	https://www.keionline.org/
	Medicines Patent Pool	https://medicinespatentpool.org/
	Médecins Sans Frontières	https://www.msf.org/
	South Centre	https://www.southcentre.int/
	Oxfam	https://www.oxfam.org/
	UICC (including ATOM)	https://www.uicc.org/
		https://www.uicc.org/atom/atom‐coalition‐home
	ESMO	https://www.esmo.org/
	Access to Medicine Foundation, including the Access to Medicine Index	https://accesstomedicinefoundation.org/
		https://accesstomedicinefoundation.org/resource/2022‐access‐to‐medicine‐index
Philanthropies	Bill and Melinda Gates Foundation	https://www.gatesfoundation.org/
	Rockefeller Foundation	https://www.rockefellerfoundation.org/
	Wellcome Trust	https://wellcome.org/
	Max Foundation	https://themaxfoundation.org/
	Ford Foundation	https://www.fordfoundation.org/
Multilateral organizations	United Nations	https://www.un.org/
	World Health Organization, including the Global Initiative for Childhood Cancer, WHO Department of Health products policy and standards	https://www.who.int/
		https://www.who.int/initiatives/the‐global‐initiative‐for‐childhood‐cancer
		https://www.who.int/teams/health‐product‐policy‐and‐standards/overview
	World Trade Organization	https://www.wto.org/
	World Intellectual Property Organization	https://www.wipo.int/
	PAHO Strategic Fund	https://www.paho.org/en/paho‐strategic‐fund
	UNICEF	https://www.unicef.org/
	UNAIDS	https://www.unaids.org/
	World Bank	https://www.worldbank.org/en/home
	UNITAIDS	https://unitaid.org/#en
Hybrid partnerships	The Global Fund	https://www.theglobalfund.org/en/
	ATOM Coalition	https://www.uicc.org/atom/atom‐coalition‐home
	St. Jude's Global Childhood Cancer Initiative (Global Platform)	https://www.stjude.org/global/collaborating‐to‐cure/global‐initiative.html
	Clinton Access Health Initiative	https://www.clintonhealthaccess.org/
	GAVI, The Vaccine Alliance	https://www.gavi.org/
Private Sector	IFPMA	https://www.ifpma.org/
	Amgen Access to Health Care	https://www.amgen.com/responsibility/healthy‐people/access‐to‐medicines/our‐approach‐to‐pricing‐access‐and‐affordability/access‐approaches‐treatment‐and‐collaborations/access‐to‐medicine‐and‐patient‐support‐programs
	Novartis Access	https://www.novartis.com/esg/access
	Gilead Global Access	https://www.gilead.com/purpose/medication‐access/global‐access
Bilateral cooperation agencies	PEPFAR	https://www.state.gov/pepfar/
	USAID	https://www.usaid.gov/
	Foreign, Commonwealth, & Development Office	https://www.gov.uk/government/organisations/foreign‐commonwealth‐development‐office
Media	Health Policy Watch	https://healthpolicy‐watch.news/
	Geneva Health Files	https://genevahealthfiles.com/

Typology for the categories of actors within this table was sourced from Frenk and Moon (2013) Governance Challenges in Global Health.

## Appendix 3

### Literature Review Search Strategy

The purpose of the literature review was to identify scholarly articles on the topic of WHO essential medicines. Since the focus of this study was on political factors, empirical studies measuring access were excluded from the analysis. Six databases were searched including OVID Embase, OVID Medline, CINAHL (nursing and allied health), PsycINFO Ovid (psychology and behavioral science, for the mental health aspect), Global Health Ovid (targeted search for developing countries). Search terms included a combination of keywords and MeSH terms that covered “World Health Organization” and “essential medicines” OR “essential drugs”.

## Appendix 4

Semistructured Interview Guide

**Table:**

**Background**	**ALL**
Could you tell me your name and current role?	
How long have you been in this position? Where did you work before?	
**Inside the EML**	**WHO**
Can you tell me about your experience at the WHO EML?	
• Significant events • Missed opportunities	
What tensions/challenges exist in advancing the goals of the EML?	
• Example? • Cancer medicines, other issues? • How has WHO addressed them?	
What data is available to monitor access?	
Funding	
**Outside of EML**	**ALL**
What is your (organizations) relationship with the WHO EML?	
• Is the list used? How?	
Who are the major actors connected to the essential medicines concept at the international level?	
• How has this evolved over the past 20 years? • Actors within global oncology?	
How collaborative are these actors?	
Which organizations/actors do you see leading the way?	
• Role of WHO/EML	
Which organizations/actors do you see opposing?	
Can you think of significant events or developments that have pushed the concept of essential medicines onto the global agenda?	
In your opinion, what are the greatest barriers to accessing essential medicines?	
• Facilitators to access essential medicines? • WHO EML? • Cancer medicines?	
**IF application to the EML**	**APPLICANTS**
Can you tell me about how the application came together?	
• Who was involved? • How was the medicine chosen?	
What is the goal of this application?	
• What role does the EML play in this goal?	
**Additional information**	**ALL**
Is there anything else you think is important?	
Is there something I should have asked you that I didn't?	
Do you have any suggestions for anyone else I should speak to?	

Stakeholders: International stakeholders connected to the essential medicines agenda

## Appendix 5

Consolidated Criteria for Reporting Qualitative Studies (COREQ): 32‐Item Checklist

**Table:**

No. Item	Guide questions/description	Reported on Page #
**Domain 1: Research team and reflexivity**		
*Personal Characteristics*		
1. Interviewer/facilitator	Which author/s conducted the interview or focus group?	Page 1
2. Credentials	What were the researcher's credentials? E.g. PhD, MD	Page 1
3. Occupation	What was their occupation at the time of the study?	Page 11
4. Gender	Was the researcher male or female?	Page 1
5. Experience and training	What experience or training did the researcher have?	Page 11
*Relationship with participants*		
6. Relationship established	Was a relationship established prior to study commencement?	NA
7. Participant knowledge of the interviewer	What did the participants know about the researcher? e.g. personal goals, reasons for doing the research	Page 11
8. Interviewer characteristics	What characteristics were reported about the interviewer/facilitator? e.g. Bias, assumptions, reasons and interests in the research topic	Page 11
**Domain 2: study design**		
*Theoretical framework*		
9. Methodological orientation and Theory	What methodological orientation was stated to underpin the study? e.g. grounded theory, discourse analysis, ethnography, phenomenology, content analysis	Page 7–9
*Participant selection*		
10. Sampling	How were participants selected? e.g. purposive, convenience, consecutive, snowball	Page 10–11
11. Method of approach	How were participants approached? e.g. face‐to‐face, telephone, mail, email	Page 11
12. Sample size	How many participants were in the study?	Page 11
13. Non‐participation	How many people refused to participate or dropped out? Reasons?	Page 11
*Setting*		
14. Setting of data collection	Where was the data collected? e.g. home, clinic, workplace	Page 12
15. Presence of non‐participants	Was anyone else present besides the participants and researchers?	Page 11
16. Description of sample	What are the important characteristics of the sample? e.g. demographic data, date	Page 11
*Data collection*		
17. Interview guide	Were questions, prompts, guides provided by the authors? Was it pilot tested?	Appendix 4
18. Repeat interviews	Were repeat interviews carried out? If yes, how many?	Page 11
19. Audio/visual recording	Did the research use audio or visual recording to collect the data?	Page 12
20. Field notes	Were field notes made during and/or after the interview or focus group?	Page 12
21. Duration	What was the duration of the interviews or focus group?	Page 12
22. Data saturation	Was data saturation discussed?	Page 11
23. Transcripts returned	Were transcripts returned to participants for comment and/or correction?	Page 12
**Domain 3: analysis and findings**		
*Data analysis*		
24. Number of data coders	How many data coders coded the data?	Page 12
25. Description of the coding tree	Did authors provide a description of the coding tree?	NA
26. Derivation of themes	Were themes identified in advance or derived from the data?	Page 12
27. Software	What software, if applicable, was used to manage the data?	Page 12
28. Participant checking	Did participants provide feedback on the findings?	Page 12
*Reporting*		
29. Quotations presented	Were participant quotations presented to illustrate the themes/findings? Was each quotation identified? e.g. participant number	Page 12
30. Data and findings consistent	Was there consistency between the data presented and the findings?	Page 12
31. Clarity of major themes	Were major themes clearly presented in the findings?	Page 12
32. Clarity of minor themes	Is there a description of diverse cases or discussion of minor themes?	Page 12

## References

[1] Wirtz VJ , Hogerzeil HV , Gray AL , et al. Essential medicines for universal health coverage. Lancet. 2017;389(10067):403‐476. 10.1016/S0140-6736(16)31599-9 27832874 PMC7159295

[2] Laing R , Waning B , Gray A , Ford N , ’t Hoen E. 25 Years of the WHO essential medicines lists: progress and challenges. Lancet. 2003;361(9370):1723‐1729. 10.1016/S0140-6736(03)13375-2 12767751

[3] Moucheraud C , Wirtz VJ , Reich MR . Evaluating the quality and use of economic data in decisions about essential medicines. Bullet World Health Organ. 2015;93(10):693‐699. 10.2471/BLT.14.149914 PMC4645430 26600611

[4] Magrini N , Robertson J , Forte G , Capello B , Moja L , Kieny MP . Tough decisions on essential medicines in 2015. Bull World Health Organ. 2015;93:283‐284. 26229193 10.2471/BLT.15.154385 PMC4431566

[5] Gray AL , Wirtz VJ , ’t Hoen EFM , Reich MR , Hogerzeil HV . Essential medicines are still essential. Lancet. 2015;386(10004):1601‐1603. 10.1016/S0140-6736(15)00514-0 26595616

[6] Brhlikova P , Persaud N , Osorio‐de‐Castro CGS , Pollock AM . Essential medicines lists are for high income countries too. BMJ. 2023;382:e076783. 10.1136/bmj-2023-076783 37669797

[7] Hogerzeil HV . The concept of essential medicines: lessons for rich countries. BMJ. 2004;329(7475):1169‐1172. 10.1136/BMJ.329.7475.1169 15539676 PMC527702

[8] Manikandan S . Are we moving towards a new definition of essential medicines? J Pharmacol Pharmacother. 2015;6(3):123‐125. 10.4103/0976-500X.162008 26311993 PMC4544131

[9] Reidenberg MM . Are we moving towards a new definition of essential medicines? J Pharmacol Pharmacother. 2015;6(4):233. 10.4103/0976-500X.171874 26816479 PMC4714396

[10] Wirtz VJ , Gray AL , Sharma S , Sun J , Hogerzeil HV . Refocusing the World Health Organization's Model List of Essential Medicines on the needs of low and middle income countries. BMJ. 2024;385:e077776. 10.1136/bmj-2023-077776 38626944

[11] Hwang TJ , Kesselheim AS , Vokinger KN . Reforming the World Health Organization's Essential Medicines List: essential but unaffordable. JAMA. 2022;328(18):1807‐1808. 10.1001/JAMA.2022.19459 36279114

[12] WHO Cancer Medicines Working Group . WHO Model Lists of Essential Medicines: Advice for the Expert Committee on Selection and Use of Essential Medicines. World Health Organization; 2023. Accessed May 7, 2024. https://cdn.who.int/media/docs/default‐source/essential‐medicines/2023‐eml‐expert‐committee/working‐group‐comments/comments_eml‐cmwg.pdf?sfvrsn=261e1d09_2

[13] Hogerzeil HV . Rare diseases and essential medicines. Int J Pharm Med. 2005;19(5):285‐288. 10.2165/00124363-200519050-00005

[14] Costa E , Moja L , Wirtz VJ , et al. Uptake of orphan drugs in the WHO essential medicines lists. Bull World Health Organ. 2024;102(1):22‐31. 10.2471/BLT.23.289731 38164340 PMC10753278

[15] Pierce GF , O'Mahony B , Kaczmarek R , et al. Risk of harm to people with haemophilia from the 2023 WHO Essential Medicines List. Lancet Haematol. 2024;11(9):e638‐e640. 10.1016/S2352-3026(24)00223-0 39116903

[16] Farrugia A . The World Health Organisation's list of essential medicines and haemophilia treatment products. Haemophilia. 2023;29(6):1387‐1389. 10.1111/hae.14879 37807613

[17] Barbui C , Purgato M . Decisions on WHO's essential medicines need more scrutiny. BMJ. 2014;349:g4798. 10.1136/BMJ.G4798 25081250

[18] Magrini N , Robertson J , de Joncheere K , Bero L . On WHO's essential medicines process and transparency. BMJ. 2014. 349:g5637. 25231094 10.1136/bmj.g5637

[19] Piggott T , Moja L , Jenei K , et al. GRADE concept 7: issues and insights linking guideline recommendations to trustworthy essential medicine lists. J Clin Epidemiol. 2024;166:111241. 10.1016/j.jclinepi.2023.111241 38123105 PMC10939133

[20] Piggott T , Moja L , Akl EA , et al. Decision criteria for selecting essential medicines and their connection to guidelines: an interpretive descriptive qualitative interview study. J Clin Epidemiol. 2023;154:146‐155. 10.1016/j.jclinepi.2022.12.007 36584732

[21] Greene JA . Making medicines essential: the emergent centrality of pharmaceuticals in global health. BioSocieties. 2011;6(1):10‐33. 10.1057/BIOSOC.2010.39

[22] Reich MR . Essential drugs: economics and politics in international health. Health Policy. 1987;8:39‐57.

[23] Sell SK , Prakash A . Using ideas strategically: the contest between business and NGO networks in intellectual property rights. Int Stud Q. 2004;48(1):143‐175.

[24] Millard C , Brhlikova P , Pollock A . Social networks and health policy: the case of misoprostol and the WHO model essential medicine list. Soc Sci Med. 2015;132:190‐196. 10.1016/j.socscimed.2015.03.011 25818380

[25] Jenei K , Glaus CEG , Vokinger KN . WHO shapes priorities for medicines? An analysis of the applicants and decision makers within the historical evolution of the WHO Model Lists of Essential Medicines. Lancet. 2024;404(10460):1365‐1374. 10.1016/S0140-6736(24)01549-6 39368844

[26] Donabedian A . Evaluating the quality of medical care. Milbank Q. 1966;44(3):166‐206. 10.2307/3348969 5338568

[27] World Health Organization. Constitution of the World Health Organization. World Health Organization; 1946. doi:12571729

[28] Guta NM . Application of Donabedian quality‐of‐care framework to assess quality of neonatal resuscitation, its outcome, and associated factors among resuscitated newborns at public hospitals of East Wollega zone, Oromia, Western Ethiopia, 2021. BMC Pediatr. 2022;22(1):605. 10.1186/s12887-022-03638-y 36258182 PMC9578212

[29] Binder C , Torres RE , Elwell D . Use of the Donabedian model as a framework for COVID‐19 response at a hospital in suburban Westchester County, New York: a facility‐level case report. J Emerg Nurs. 2021;47(2):239‐255. 10.1016/j.jen.2020.10.008 33317860 PMC7831996

[30] Lawson EF , Yazdany J . Healthcare quality in systemic lupus erythematosus: using Donabedian's conceptual framework to understand what we know. Int J Clin Rheumtol. 2012;7(1):95‐107. 10.2217/ijr.11.65 22448191 PMC3308354

[31] Saheb T , Saheb T . Digital health policy decoded: mapping national strategies using Donabedian's model. Health Policy. 2024;147:105134. 10.1016/j.healthpol.2024.105134 39053416

[32] Ghofrani M , Valizadeh L , Zamanzadeh V , Ghahramanian A , Janati A , Taleghani F . Adapting the Donabedian model in undergraduate nursing education: a modified Delphi study. BMC Med Educ. 2024;24(1):202. 10.1186/s12909-024-05187-7 38413915 PMC10900582

[33] LoPorto J . Application of the Donabedian quality‐of‐care model to New York State direct support professional core competencies: how structure, process, and outcomes impacts disability services. JOSC. 2020;12(1). 10.5590/JOSC.2020.12.1.05

[34] Lasswell HD . Politics: Who Gets What, When, How. Pickle Partners Publishing; 1950.

[35] Gauvin FP . Understanding Policy Developments and Choices Through the “3‐i” Framework: Interests, Ideas and Institutions. National Collaborating Centre for Healthy Public Policy; 2014.

[36] Shearer JC , Abelson J , Kouyaté B , Lavis JN , Walt G . Why do policies change? Institutions, interests, ideas and networks in three cases of policy reform. Health Policy Plan. 2016;31(9):1200‐1211. 10.1093/heapol/czw052 27233927 PMC5886035

[37] Hall PA . Policy paradigms, social learning, and the state: the case of economic policymaking in Britain. Comp Polit. 1993;25(3):275‐296. 10.2307/422246

[38] Koon AD , Hawkins B , Mayhew SH . Framing and the health policy process: a scoping review. Health Policy Plan. 2016;31(6):801‐816. 10.1093/heapol/czv128 26873903 PMC4916318

[39] North DC. Institutions. J Econ Perspect. 1991;5(1):97‐112.

[40] Murphy M . Interests, institutions and ideas: explaining Irish social security policy. Policy Press. 2012;40(3):347‐365. 10.1332/030557312X626640

[41] Buse K , Dickinson C , Sidibé M . HIV: know your epidemic, act on its politics. J R Soc Med. 2008;101(12):572‐573. 10.1258/jrsm.2008.08k036 19092021 PMC2625375

[42] Walt G , Shiffman J , Schneider H , Murray SF , Brugha R , Gilson L . “Doing” health policy analysis: methodological and conceptual reflections and challenges. *Health Policy Plan* . 2008;23(5):308‐317. 10.1093/heapol/czn024 PMC2515406 18701552

[43] World Health Organization . Publications database. 2024. Accessed August 1, 2024. https://www.who.int/publications

[44] Watal J , Taubman A , eds. The Making of the TRIPS Agreement: Personal Insights from the Uruguay Round Negotiations. World Trade Organization; 2015.

[45] Velásquez G , Correa CM , Ido VHP . Intellectual Property, Human Rights and Access to Medicines: A Selected and Annotated Bibliography. 3rd ed. South Centre; 2020.

[46] Velásquez G , Boulet P . The WHO “Red Book” on Access to Medicines and Intellectual Property: 20 Years Later. South Centre; 2015.

[47] Velásquez G , Boulet P . Globalization and Access to Drugs: Implications of the WTO/TRIPS Agreement. 2nd ed. World Health Organization; 1999. Accessed February 26, 2024. https://web.archive.org/web/20061211134310/http://www.who.int/medicines/areas/policy/who‐dap‐98‐9rev.pdf

[48] Velásquez G . Intellectual property and access to medicines and vaccines. In: Velásquez G , ed. Vaccines, Medicines and COVID‐19: How Can WHO Be Given a Stronger Voice? Springer International Publishing; 2022:73‐92. *SpringerBriefs in Public Health*; South Centre Training Paper 1. 10.1007/978-3-030-89125-1_5

[49] Velásquez G . Some Critical Issues Related to Access to Medicines and Intellectual Property. South Centre; 2014. Accessed February 7, 2025. https://www.southcentre.int/wp‐content/uploads/2016/05/Bk_2014_Some‐Critical‐Issues‐Related‐to‐Access‐to‐Medicines‐and‐IP_EN.pdf

[50] Velásquez G . Access to Medicines and Intellectual Property: the Contribution of the World Health Organization. South Centre; 2013. Accessed February 7, 2025. https://www.southcentre.int/wp‐content/uploads/2013/05/RP47_WTO‐role‐in‐IP‐and‐access‐to‐medicines_EN.pdf

[51] Turner M . The global governance of HIV/AIDS: Intellectual property and access to essential medicines. Glob Public Health. 2014;9(9):1117‐1118. 10.1080/17441692.2014.955042

[52] ’t Hoen E . The Global Politics of Pharmaceutical Monopoly Power. AMB; 2009.

[53] ’t Hoen E . Report of the Commission on Intellectual Property Rights, Innovation and Public Health: a call to governments. Bull World Health Organ. 2006;84(5):421‐423. 16710558 10.2471/blt.06.032391 PMC2627339

[54] Shadlen KC . The political economy of pharmaceutical patents. In: Shadlen KC , ed. Coalitions and Compliance: The Political Economy of Pharmaceutical Patents in Latin America. Oxford University Press; 2017:28‐60. 10.1093/oso/9780199593903.003.0002

[55] Loff B . Patents and access to essential drugs. Trans R Soc Trop Med Hyg. 2003;97(1):6‐9. 10.1016/S0035-9203(03)90002-2 12886793

[56] Legge DG . Intellectual property, innovation and public health at WHO: a chronology. WHO Tracker. Updated January 10, 2022. Accessed February 7, 2025. https://who‐track.phmovement.org/ipandinnovation

[57] ’ t Hoen E TRIPS , pharmaceutical patents, and access to essential medicines: a long way from Seattle to Doha. In: Kirton JJ , ed. Global Health. 1st ed. Routledge; 2009;304‐324. 10.4324/9781315254227 15709298

[58] Ho CM . A history of access to medicine through the lens of patent perspectives. In: Ho CM , ed. Access to Medicine in the Global Economy: International Agreements on Patents and Related Rights. Oxford University Press; 2011:325‐354. 10.1093/acprof:oso/9780195390124.003.0012

[59] Gleeson D , Lexchin J , Labonté R , et al. Analyzing the impact of trade and investment agreements on pharmaceutical policy: provisions, pathways and potential impacts. Global Health. 2019;15(1):1‐17. 10.1186/S12992-019-0518-2/TABLES/2 31775767 PMC6882307

[60] Beall RF , Kuhn R , Attaran A . Compulsory licensing often did not produce lower prices for antiretrovirals compared to international procurement. Health Aff (Millwood). 2015; 34(3):493‐501. 10.1377/hlthaff.2014.0658 25732501

[61] Lorgen CC . Dancing with the state: the role of NGOs in health care and health policy. J Int Dev. 1998;10(3):323‐339. 10.1002/(SICI)1099-1328(199805/06)10:3<323::AID-JID424>3.0.CO;2-4

[62] Kay A , Williams OD , eds. Global Health Governance: Crisis, Institutions and Political Economy. Palgrave Macmillan UK; 2009. 10.1057/9780230249486

[63] Horton R . Offline: global health and the private sector. Lancet. 2018;391(10136):2196. 10.1016/S0140-6736(18)31253-4 29893214

[64] Gómez EJ . Civil society in global health policymaking: a critical review. Global Health. 2018;14(1):73. 10.1186/s12992-018-0393-2 30045738 PMC6060457

[65] Buse K , Lee K . Business and Global Health Governance. Discussion Paper No. 5. Centre on Global Change and Health London School of Hygiene and Tropical Medicine; Department of Ethics, Trade, Human Rights, and Health Law, World Health Organization; 2006.

[66] Brass JN , Longhofer W , Robinson RS , Schnable A . NGOs and international development: a review of thirty‐five years of scholarship. World Dev. 2018;112:136‐149. 10.1016/j.worlddev.2018.07.016

[67] Abraham J . The pharmaceutical industry as a political player. Lancet. 2002;360(9344):1498‐1502. 12433532 10.1016/S0140-6736(02)11477-2

[68] Barnett M , Duvall R . Power in international politics. Int Organ. 2005;59(1):39‐75.

[69] Collins K . Profitable gifts: a history of the Merck Mectizan donation program and its implications for international health. Perspect Biol Med. 2004;47(1):100‐109. 10.1353/pbm.2004.0004 15061171

[70] Comanor WS . The political economy of the pharmaceutical industry. J Econ Lit. 1986;24(3):1178‐1217. 11617316

[71] Collier J , Iheanacho I . The pharmaceutical industry as an informant. Lancet. 2002;360(9343):1405‐1409. 10.1016/S0140-6736(02)11394-8 12424005

[72] Hardon A . New WHO leader should aim for equity and confront undue commercial influences. Lancet. 2003;361(9351):6. 10.1016/S0140-6736(03)12177-0 12521037

[73] Hardwicke CJ . The World Health Organization and the pharmaceutical industry. Adv Drug React Toxicol Rev. 2002;21(1):51‐99. 10.1007/BF03256183 12140907

[74] Harman S . The Bill and Melinda Gates Foundation and legitimacy in global health governance. Glob Gov. 2016;22(3):349‐368. 10.1163/19426720-02203004

[75] ’t Hoen EFM , Hogerzeil HV , Quick JD , Sillo HB . A quiet revolution in global public health: the World Health Organization's Prequalification of Medicines Programme. J Public Health Policy. 2014;35(2):137‐161. doi: 10.1057/jphp.2013.53 24430804

[76] Perehudoff SK , Toebes B , Hogerzeil H . Essential medicines in national constitutions. Health Hum Rights. 2016;18(1):141‐156. 27781006 PMC5070687

[77] Ruggie JG . Multinationals as global institution: power, authority and relative autonomy. Regul Gov. 2018;12(3):317‐333. 10.1111/rego.12154

[78] Sismondo S . Access to medicines, access to markets. Front Sociol. 2020;5:58. 10.3389/fsoc.2020.00058 33869464 PMC8022690

[79] Simão M , Wirtz VJ , Al‐Ansary LA , et al. A global accountability mechanism for access to essential medicines. Lancet. 2018;392(10163):2418‐2420. 10.1016/S0140-6736(18)32986-6 30527401

[80] Samsky A . Scientific sovereignty: how international drug donation programs reshape health, disease, and the state. Cult Anthropol. 2012;27(2):310‐332. 10.1111/j.1548-1360.2012.01145.x 22737726

[81] Shiffman J . Donor funding priorities for communicable disease control in the developing world. Health Policy Plan. 2006;21(6):411‐420. 10.1093/heapol/czl028 16984894

[82] Shiffman J . Knowledge, moral claims and the exercise of power in global health. Int J Health Policy Manag. 2014;3(6):297‐299. doi: 10.15171/ijhpm.2014.120 25396204 PMC4226618

[83] Moon S . Power in global governance: an expanded typology from global health. Global Health. 2019;15(Suppl 1):74. 10.1186/s12992-019-0515-5 31775779 PMC6881906

[84] Hogerzeil HV . Big pharma and social responsibility — the access to medicine index. N Engl J Med. 2013;369(10):896‐899. 10.1056/NEJMp1303723 24004116

[85] Hogerzeil HV , Iyer JK , Urlings L , Prasad T , Brewer S . Is the pharmaceutical industry improving with regard to access to essential medicines? Lancet Glob Health. 2014;2(3):e139‐e140. 10.1016/S2214-109X(13)70159-1 25102843

[86] Mhazo AT , Maponga CC . Framing access to essential medicines in the context of Universal Health Coverage: a critical analysis of health sector strategic plans from eight countries in the WHO African region. BMC Health Serv Res. 2022;22(1):1390. 10.1186/s12913-022-08791-9 36419062 PMC9682662

[87] Barlow P , Thow AM . Neoliberal discourse, actor power, and the politics of nutrition policy: a qualitative analysis of informal challenges to nutrition labelling regulations at the World Trade Organization, 2007–2019. Soc Sci Med. 2021;273:113761. 10.1016/j.socscimed.2021.113761 33621752

[88] Adekoya I , Maraj D , Steiner L , et al. Comparison of antibiotics included in national essential medicines lists of 138 countries using the WHO Access, Watch, Reserve (AWaRe) classification: a cross‐sectional study. Lancet Infect Dis. 2021;21(10):1429‐1440. 10.1016/S1473-3099(20)30854-9 34332706

[89] Costa E . Rare diseases & model list of essential medicines. PowerPoint presented at: 24th Meeting of the WHO Expert Committee on the Selection and Use of Essential Medicines; April 24, 2023. Geneva, Switzerland. Accessed October 3, 2023. https://cdn.who.int/media/docs/default‐source/essential‐medicines/2023‐eml‐expert‐committee/open‐session/invited‐presentations/os_costa.pdf?sfvrsn=5ec440a_1

[90] Brhlikova P , Deivanayagam TA , Babar ZUD , Osorio‐de‐Castro CGS , Caetano R , Pollock AM . Essential medicines concept and health technology assessment approaches to prioritising medicines: selection versus incorporation. J Pharm Policy Pract. 2023;16(1):88. 10.1186/s40545-023-00595-4 37443124 PMC10347820

[91] Das P , Horton R . Essential medicines for universal health coverage. Lancet. 2017;389(10067):337‐339. 10.1016/S0140-6736(16)31907-9 27832878

[92] Persaud N , Jiang M , Shaikh R , et al. Comparison of essential medicines lists in 137 countries. Bullet World Health Organ. 2019;97(6):394‐404C. 10.2471/BLT.18.222448 PMC6560372 31210677

[93] Bazargani YT , Ewen M , De Boer A , Leufkens HGM , Mantel‐Teeuwisse AK. Essential medicines are more available than other medicines around the globe. PLoS One. 2014;9(2):e87576. 10.1371/JOURNAL.PONE.0087576 24533058 PMC3922716

[94] Breckenridge A . Debate that “This house believes the essential drug concept hinders the effective deployment of drugs in developing countries.” Trans R Soc Trop Med Hyg. 2003;97(1):1. 10.1016/S0035-9203(03)90000-9 12886791

[95] Bigdeli M , Jacobs B , Tomson G , et al. Access to medicines from a health system perspective. Health Policy Plan. 2013;28(7):692‐704. 10.1093/HEAPOL/CZS108 23174879 PMC3794462

[96] Brundtland GH . Essential medicines: 25 years of better health. JAMA. 2002;288(24):3102. 10.1001/jama.288.24.3102 12495381

[97] Brundtland GH . Address by Dr Gro Harlem Brundtland, Director‐General, to the fifty‐fifth World Health Assembly, Geneva, Monday, 13 May 2002. World Health Organization. May 13, 2002. Accessed February 26, 2024. https://apps.who.int/gb/ebwha/pdf_files/WHA55/ea553.pdf

[98] Cassels A . WHO publishes world's first formulary for essential drugs. CMAJ. 2002;167(11):1278.12451093

[99] Di Cesare M , Jarvis JD , Scarlatescu O , et al. NOACs Added to WHO's Essential Medicines List: recommendations for future policy actions. Glob Heart. 2020;15(1):67. 10.5334/gh.774 33150132 PMC7546116

[100] Chattu VK , Singh B , Pattanshetty S , Reddy S . Access to medicines through global health diplomacy. Health Promot Perspect. 2023;13(1):40‐46. doi: 10.34172/hpp.2023.05 37309432 PMC10257564

[101] Chirac P , Laing R . Updating the WHO essential drugs list. Lancet. 2001;357(9262):1134. 10.1016/S0140-6736(00)04291-4 11303617

[102] Cohen D . Roche asks WHO to remove Avastin from Essential Medicines List. BMJ. 2017;356:j779. 10.1136/bmj.j779 28193603

[103] De Lima L . International Association for Hospice and Palliative Care list of essential medicines for palliative care. Ann Oncol. 2007;18(2):395‐399. 10.1093/annonc/mdl373 17071936

[104] Denburg A , Arora B , Arora RS , et al. Access to essential medicines for children with cancer: a joint SIOP‐CCI position statement. Lancet Oncol. 2017;18(1):20‐22. 10.1016/S1470-2045(16)30652-0 28049570

[105] Dugani S , Wasan KM , Kissoon N . World Health Organization and essential medicines. J Pharm Sci. 2018;107(5):1261‐1262. 10.1016/j.xphs.2017.12.019 29277641

[106] Etienne CF . Diabetes and the WHO Model List of Essential Medicines. Lancet Diabetes Endocrinol. 2022;10(1):21. 10.1016/S2213-8587(21)00317-X 34919868

[107] Ford N , Piédagnel JM . WHO must continue its work on access to medicines in developing countries. Lancet. 2003;361(9351):3. 10.1016/S0140-6736(03)12173-3 12521032

[108] Forman L , Parker M . Should COVID‐19 vaccines authorized for emergency use be considered “essential” medicines? Health Hum Rights. 2021;23(1):145‐150. 34194208 PMC8233020

[109] Furlow B . WHO Essential Medicines Committee spotlights unaffordable drugs. Lancet Oncol. 2021;22(11):1503. 10.1016/S1470-2045(21)00575-1 34627500

[110] Gibson L . WHO puts abortifacients on its essential drug list. BMJ. 2005;331(7508):68. 10.1136/bmj.331.7508.68-c PMC558642 16002864

[111] Gill R , Ganatra B , Althabe F . WHO essential medicines for reproductive health. BMJ Global Health. 2019;4(6):e002150. 10.1136/bmjgh-2019-002150 PMC6936467 31908878

[112] Global Diagnostics Alliance; Global Medical Technology Alliance . Letter to Dr. Suzanne Hill, Director, Essential Medicines and Health Products Department, World Health Organization. World Health Organization. March 10, 2019. Accessed March 14, 2024. https://cdn.who.int/media/docs/default‐source/in‐vitro‐diagnostics/edl/second‐meeting/07_gda_gmta_comment_letter.pdf?msclkid=1feee30cd01011ec9949e2b579e51379

[113] Gray NJ , Chanoine JP , Farmer MY , et al. NCDs and the WHO Essential Medicines Lists: children need universal health coverage too. Lancet Child Adolesc Health. 2019;3(11):756‐757. 10.1016/S2352-4642(19)30294-9 31537467

[114] Gupta S . Expanding the WHO list of essential medicines for children: a call for further action. Pediatr Blood Cancer. 2015;62(10):1685‐1686. 10.1002/pbc.25597 26132995

[115] Helling‐Borda M . Memories of the first expert committee meeting and celebrating 25 years later. Essent Drugs Monit. 2003;32:14‐15.

[116] Helling‐Borda M ; WHO Action Programme on Essential Drugs. The Essential Drugs Concept and Its Implementation. World Health Organization; 1985. Accessed July 7, 2023. https://apps.who.int/iris/handle/10665/58182

[117] Hogerzeil HV . Opposing the motion. Trans R Soc Trop Med Hyg. 2003;97(1):14‐15. 10.1016/S0035-9203(03)90005-8 12886796

[118] Holloway KA , Henry D . WHO essential medicines policies and use in developing and transitional countries: an analysis of reported policy implementation and medicines use surveys. PLoS Med. 2014;11(9):e1001724. 10.1371/journal.pmed.1001724 25226527 PMC4165598

[119] Holloway KA , Rosella L , Henry D . The impact of WHO essential medicines policies on inappropriate use of antibiotics. PLoS One. 2016;11(3):e0152020. 10.1371/journal.pone.0152020 27002977 PMC4803297

[120] Hutchings J , Neroutsos K , Donnelly K . Making the list: the role of essential medicines lists in reproductive health. Int Perspect Sex Reprod Health. 2010;36(04):205‐208. 10.1363/3620510 21245027

[121] Joshi TP , Ren V . Essentiality and economy: a feasibility approach to evaluating suggested revisions to the World Health Organization Model List of Essential Medicines for skin disease. Br J Dermatol. 2021;185(5):1077. 10.1111/bjd.20565 34105761

[122] Kamerman PR , Wadley AL , Davis KD , et al. World Health Organization essential medicines lists: where are the drugs to treat neuropathic pain? Pain. 2015;156(5):793‐797. 10.1097/01.j.pain.0000460356.94374.a1 25894010 PMC4670621

[123] Kishore S , Herbstman B . Adding a medicine to the WHO Model List of Essential Medicines. Clin Pharmacol Ther. 2009;85(3):237‐239. 10.1038/clpt.2008.258 19223877 PMC8052985

[124] Kishore SP , Blank E , Heller DJ , et al. Modernizing the World Health Organization List of Essential Medicines for preventing and controlling cardiovascular diseases. J Am Coll Cardiol. 2018;71(5):564‐574. 10.1016/J.JACC.2017.11.056 29406862

[125] Kishore SP , Bitton A , Cravioto A , Yach D . Enabling access to new WHO essential medicines: the case for nicotine replacement therapies. Global Health. 2010;6(1):22. 10.1186/1744-8603-6-22 21092092 PMC2994846

[126] Rimmer K , Shah H , Thakur K . Expanding medicines for neurologic disorders on the WHO Model List. Neurology. 2017;88(10):e87‐e91. 10.1212/WNL.0000000000003691 28265046

[127] Robertson J , Hill SR . The Essential Medicines List for a global patient population. Clin Pharmacol Ther. 2007;82(5):498‐500. 10.1038/SJ.CLPT.6100392 17952104

[128] Rundle CW , Fortugno AP , Maghfour J , et al. Evaluating the World Health Organization Model List of Essential Medicines for skin disease. Br J Dermatol. 2021;185(2):451‐453. 10.1111/BJD.20081 33764503

[129] Russ K . All documents from Freedom of Information Act Request USTR_FY21‐87. Zenodo. May 18, 2022. Accessed March 14, 2024. https://zenodo.org/records/6525568

[130] Russ KN , Baker P , Kang M , McCoy D . Corporate lobbying on US positions toward the World Health Organization. J Glob Health. 2022;17(1):37‐83.

[131] Samukange WT , Gardarsdottir H , Leufkens HGM , Mantel‐Teeuwisse AK . Selection of blood, blood components, and blood products as essential medicines in 105 low‐ and middle‐income countries. Transfus Med Rev. 2020;34(2):94‐100. 10.1016/J.TMRV.2019.10.005 31761652

[132] Sen P , Kohli M , Pai M . Industry perspectives on the WHO Essential Diagnostics List. J Clin Microbiol. 2019;57(2):e01637‐18. 10.1128/JCM.01637-18 30518544 PMC6355519

[133] Seuba X . A human rights approach to the WHO Model List of Essential Medicines. Bull World Health Organ. 2006;84(5):405‐411. 10.2471/BLT.04.019133 16710552 PMC2627340

[134] Syrett K . Essential but expensive? The world health organization, access to medicines and human rights. Neth Q Hum Rights. 2019;37(2):139‐156. 10.1177/0924051919844373

[135] Sridhar D . Improving access to essential medicines: how health concerns can be prioritised in the global governance system. Public Health Ethics. 2008;1(2):83‐88. 10.1093/phe/phn012 19461853 PMC2591098

[136] Stolk P , Willemen MJC , Leufkens HGM . “Rare essentials”: drugs for rare diseases as essential medicines. Bullet World Health Organ. 2006;84(9):745‐751. 10.2471/BLT.06.031518 PMC2627463 17128345

[137] ’t Hoen E , Meyer S , Bannenberg P , Perehudoff K , Reed T , Barber MJ . Improving affordability of new essential cancer medicines. Lancet Oncol. 2019;20(8):1052‐1054. 10.1016/S1470-2045(19)30459-0 31303433

[138] Barber MJ. A.40 Risdiplam ‐ spinal muscular atrophy ‐ EML and EMLc. April 6, 2023. Accessed February 12, 2024. https://cdn.who.int/media/docs/default‐source/essential‐medicines/2023‐eml‐expert‐committee/public‐comments/a40_risdiplam_barber.pdf?sfvrsn=28a26d21_1

[139] Mishra SR , Li SWS , Onarheim KH , et al. Young people have a new vision for essential medicines. Lancet Diabetes Endocrinol. 2016;4(9):733‐734. 10.1016/S2213-8587(16)30153-X 27460306

[140] Gotham D , Onarheim KH , Barber MJ . How the MDGs gave up on measuring access to medicines. Lancet Glob Health. 2016;4(5):e296‐e297. 10.1016/S2214-109X(16)00066-8 27102189

[141] The Lancet Global Health . Essential diagnostics: mind the gap. Lancet Glob Health. 2021;9(11):e1474. 10.1016/S2214-109X(21)00467-8 34678179 PMC8654090

[142] Experts in Chronic Myeloid Leukemia . The price of drugs for chronic myeloid leukemia (CML) is a reflection of the unsustainable prices of cancer drugs: from the perspective of a large group of CML experts. Blood. 2013;121(22):4439‐4442. 10.1182/blood-2013-03-490003 23620577 PMC4190613

[143] Zanichelli V , Sharland M , Cappello B , et al. The WHO AWaRe (Access, Watch, Reserve) antibiotic book and prevention of antimicrobial resistance. Bull World Health Organ. 2023;101(4):290‐296. 10.2471/BLT.22.288614

[144] Antezana FS . Essential drugs – whose responsibility? J R Soc Med. 1981;74(3):175‐177. 10.1177/014107688107400301 7205852 PMC1438274

[145] Atif M , Malik I , Dawoud D , Gilani A , Ahmed N , Babar ZUD . Essential medicine list, policies, and the World Health Organization. Encycl Pharm Pract Clin Pharm. 2019;1:239‐249. 10.1016/B978-0-128-12735-3.00061-3

[146] Eniu A , Torode J , Magrini N , Bricalli G . Back to the essence of medical treatment in oncology: the 2015 WHO Model List of Essential Medicines. ESMO Open. 2016;1(2):e000030. 10.1136/esmoopen-2015-000030 27843592 PMC5070297

[147] IMS Institute for Healthcare Informatics . Understanding the Role and Use of Essential Medicines Lists. IMS Institute for Healthcare Informatics; 2015. Accessed August 11, 2023. https://www.farmaindustria.es/web_en/wp‐content/uploads/sites/3/2015/04/IIHI_Essential_Medicines_Report_2015‐Web3.pdf

[148] Bai L , Zhan Y , Zhou Y , et al. Evidence of clinical benefit of WHO essential anticancer medicines for children, 2011–2021. eClinicalMedicine. 2023;59:101966. 10.1016/j.eclinm.2023.101966 37125406 PMC10130597

[149] Hill S , Pang T . Leading by example: a culture change at WHO. Lancet. 2007;369(9576):1842‐1844. 10.1016/S0140-6736(07)60676-X 17493675

[150] Welch C . The composition of WHO's expert committee on essential medicines needs more scrutiny. BMJ. 2014;349:g5211. 10.1136/bmj.g5211 25138008

[151] Burrone E , Gotham D , Gray A , et al. Patent pooling to increase access to essential medicines. Bullet World Health Organ. 2019;97(8):575‐577. 10.2471/BLT.18.229179 PMC6653814 31384076

[152] Beckmann MN , Hall RL . Elite interviewing in Washington, DC. In: Mosley L , ed. Interview Research in Political Science. Cornell University Press; 2013:196‐208. Accessed September 19, 2023. https://www.jstor.org/stable/10.7591/j.ctt1xx5wg.15

[153] Executive Board 109 . WHO Medicines Strategy: Revised Procedure for Updating WHO's Model List of Essential Drugs: Report by the Secretariat . World Health Organization; 2002. Accessed April 2, 2023. https://apps.who.int/iris/handle/10665/78389

[154] Magrini N , Moja L , Cappello B , De Joncheere K , Forte G . The 19th EML as a global standard: an innovative approach or just an opportunity for new and effective medicines? J Pharm Policy Pract. 2015;8(Suppl 1):O14. 10.1186/2052-3211-8-S1-O14

[155] Jarvis JD , Murphy A , Perel P , Persaud N . Acceptability and feasibility of a national essential medicines list in Canada: a qualitative study of perceptions of decision‐makers and policy stakeholders. CMAJ. 2019;191(40):E1093‐E1099. 10.1503/cmaj.190567 31591095 PMC6779536

[156] World Health Organization . Revising the Procedures for Updating WHO's Model Lists of Essential Medicines: Consultation Report, Geneva, Switzerland, 2–3 November 2023; World Health Organization; 2024. Accessed February 7, 2025. https://www.who.int/publications/i/item/9789240091696

[157] Iwunna O , Kennedy J , Harmer A . Flexibly funding WHO? An analysis of its donors’ voluntary contributions. BMJ Global Health. 2023;8(4):e011232. 10.1136/bmjgh-2022-011232 PMC10083790 37024117

[158] World Health Organization . WHO Medicines, Vaccines, and Pharmaceuticals Annual Report 2018: Promoting Access to Safe, Effective, Quality and Affordable Essential Medical Products for All . World Health Organization; 2019. Accessed December 15, 2023. https://iris.who.int/bitstream/handle/10665/324765/WHO‐MVP‐EMP‐2019.03‐eng.pdf?sequence=1

[159] World Health Organization . WHO Essential Medicines & Health Products Annual Report 2017: Towards Access 2030 . World Health Organization; 2018. Accessed December 15, 2023. https://iris.who.int/bitstream/handle/10665/272972/WHO‐EMP‐2018.01‐eng.pdf?ua=1

[160] World Health Organization . WHO Essential Medicines & Health Products Annual Report 2016 . World Health Organization; 2016. Accessed December 15, 2023. https://web.archive.org/web/20171014010318/https://www.who.int/medicines/publications/annual‐reports/WHO_EMP_Report_2016_Online.pdf

[161] World Health Organization . WHO Essential Medicines and Health Products Annual Report 2015 . World Health Organization; 2016. Accessed December 15, 2023. https://iris.who.int/bitstream/handle/10665/252838/WHO‐EMP‐2016.02‐eng.pdf?sequence=1

[162] Kudymowa J , Hu J , Hird T . An Overview of the WHO Essential Medicines List: Procedures, Usage, and Potential Improvements. Global Health and Development; Global Health Interventions; 2023. Accessed June 5, 2023. https://rethinkpriorities.org/publications/who‐essential‐medicines‐list

[163] MS International Federation . Key Steps to Get Started in Using the WHO EML in Your Advocacy Efforts. YouTube. October 9, 2023. Accessed January 11, 2024. https://www.youtube.com/watch?v=YkAVcFWZBcU

[164] International Federation of Pharmaceutical Manufactorers & Associations . IFPMA Position Paper on the WHO Essential Medicines List . World Health Organization; 2016. Accessed September 19, 2023. https://www.ifpma.org/wp‐content/uploads/2023/01/i2023_EML‐PP_Mar2019.pdf

[165] Roemer‐Mahler A . Business conflict and global politics: the pharmaceutical industry and the global protection of intellectual property rights. Rev Int Polit Econ. 2013;20(1):121‐152. 10.1080/09692290.2011.645848

[166] World Health Organization Expert Committee . The Selection and Use of Essential Medicines. World Health Organization; 2005. *WHO Technical Report Series*; no 933.

[167] Chirac P . Translating the essential drugs concept into the context of the year 2000. Trans R Soc Trop Med Hyg. 2003;97(1):10‐12. 10.1016/S0035-9203(03)90003-4 12886794

[168] Basu S , Brown C , Beran D , et al. Expanding access to newer medicines for people with type 2 diabetes mellitus in low‐ and middle‐income countries: a microsimulation and price target analysis. Lancet Diabetes Endocrinol. 2021;9(12):825‐836. 10.1016/S2213-8587(21)00240-0 34656210

[169] World Health Organization Expert Committee . The Selection and Use of Essential Medicines. World Health Organization; 2013. *WHO Technical Report Series*; no 985. 121177

[170] Shulman LN , Wagner CM , Barr R , et al. Proposing essential medicines to treat cancer: methodologies, processes, and outcomes. J Clin Oncol. 2016;34(1):69‐75. 10.1200/JCO.2015.61.8736 26578613 PMC5070565

[171] World Health Organization Expert Committee on Selection and Use of Essential Medicines . The Selection and Use of Essential Medicines. World Health Organization; 2023. *WHO Technical Report Series*; no 1049. Accessed May 3, 2024. https://iris.who.int/bitstream/handle/10665/376570/9789240089266‐eng.pdf?sequence=1

[172] MSF responds to publication of new edition of WHO Model List of Essential Medicines . Médecins Sans Frontières Access Campaign. May 12, 2015. Accessed August 7, 2024. https://www.msfaccess.org/pt‐br/msf‐responds‐publication‐new‐edition‐who‐model‐list‐essential‐medicines

[173] Hogerzeil HV , Walker GJ , Sallami AO , Fernando G . Impact of an essential drugs programme on availability and rational use of drugs. Lancet. 1989;1(8630):141‐142. 10.1016/s0140-6736(89)91152-5 2563055

[174] Health Action International; World Health Organization . Measuring Medicine Prices, Availability, Affordability and Price Components. 2nd ed. World Health Organization; 2008. Accessed October 17, 2023. https://haiweb.org/what‐we‐do/price‐availability‐affordability/collecting‐evidence‐on‐medicine‐prices‐availability/

[175] About. Global Essential Medicines . 2017. Accessed August 1, 2024. https://global.essentialmeds.org/about

[176] Piggott T , Nowak A , Brignardello‐Petersen R , et al. Global status of essential medicine selection: a systematic comparison of national essential medicine lists with recommendations by WHO. BMJ Open. 2022;12(2):e053349. 10.1136/BMJOPEN-2021-053349 PMC8845216 35144950

[177] Jarvis JD , Woods H , Bali A , Oronsaye E , Persaud N . Selection of WHO‐recommended essential medicines for non‐communicable diseases on national essential medicines lists. PLoS One. 2019;14(8):e0220781. 10.1371/JOURNAL.PONE.0220781 31398195 PMC6688805

[178] Taglione MS , Persaud N . Assessing variation among the national essential medicines lists of 21 high‐income countries: a cross‐sectional study. BMJ Open. 2021;11(8):45262. 10.1136/BMJOPEN-2020-045262 PMC8359480 34380717

[179] Jenei K , Wirtz VJ . Measuring access to essential medicines in the sustainable development goals. Bull World Health Organ. 2024;102(8):555‐555A. 10.2471/BLT.24.291399 39091967 PMC11276153

[180] Bortone B , Jackson C , Hsia Y , Bielicki J , Magrini N , Sharland M . High global consumption of potentially inappropriate fixed dose combination antibiotics: analysis of data from 75 countries. PLoS One. 2021;16(1):e0241899. 10.1371/JOURNAL.PONE.0241899 33471786 PMC7817037

[181] Hsia Y , Lee BR , Versporten A , et al. Use of the WHO Access, Watch, and Reserve classification to define patterns of hospital antibiotic use (AWaRe): an analysis of paediatric survey data from 56 countries. Lancet Glob Health. 2019;7(7):e861‐e871. 10.1016/S2214-109X(19)30071-3 31200888

[182] Sharland M , Cappello B , Ombajo LA , et al. The WHO AWaRe Antibiotic Book: providing guidance on optimal use and informing policy. Lancet Infect Dis. 2022;22(11):1528‐1530. 10.1016/S1473-3099(22)00683-1 36309019

[183] Mudenda S , Daka V , Matafwali SK . World Health Organization AWaRe framework for antibiotic stewardship: where are we now and where do we need to go? An expert viewpoint. Antimicrob Steward Healthc Epidemiol. 2023;3(1):e84. 10.1017/ash.2023.164 37179758 PMC10173285

[184] Stone RW . Controlling Institutions: International Organizations and the Global Economy. 1st ed. Cambridge University Press; 2011. 10.1017/CBO9780511793943

[185] Piggott T , Moja L , Huttner B , et al. WHO model list of essential medicines: visions for the future. Bull World Health Organ. 2024;102(10):722‐729. 10.2471/BLT.24.292359 39318894 PMC11418853

[186] World Health Organization . Pricing of Cancer Medicines and Its Impacts. World Health Organization; 2018. Accessed November 9, 2021 . https://iris.who.int/bitstream/handle/10665/277190/9789241515115‐eng.pdf?ua=1

[187] Jenei K , Aziz Z , Booth C , et al. Cancer medicines on the WHO Model List of Essential Medicines: processes, challenges, and a way forward. Lancet Glob Health. 2022;10(12):e1860‐e1866. 10.1016/s2214-109x(22)00376-x 36183737

[188] Horton, J. Proposing the motion. Trans R Soc Trop Med Hyg. 2003; 97(1):12‐13. 10.1016/s0035-9203(03)90004-6 12886795

